# Characterizing people with frequent emergency department visits and substance use: a retrospective cohort study of linked administrative data in Ontario, Alberta, and B.C., Canada

**DOI:** 10.1186/s12873-022-00673-x

**Published:** 2022-07-14

**Authors:** Jessica Moe, Yueqiao Elle Wang, Michael J. Schull, Kathryn Dong, Margaret J. McGregor, Corinne M. Hohl, Brian R. Holroyd, Kimberlyn M. McGrail

**Affiliations:** 1 Department of Emergency Medicine, University of British Columbia, Diamond Health Care Centre, 11th Floor – 2775 Laurel Street, Vancouver, BC V5Z 1M9 Canada; 2 Department of Emergency Medicine, Vancouver General Hospital, 920 West 10th Avenue, Vancouver, BC V5Z 1M9 Canada; 3 BC Centre for Disease Control, 655 West 12th Avenue, Vancouver, BC V5Z 4R4 Canada; 4 Institute for Clinical Evaluative Sciences, G1 06, 075 Bayview Avenue, Toronto, ON M4N 3M5 Canada; 5 Department of Medicine, University of Toronto, 27 King’s College Circle, Toronto, ON M5S 1A1 Canada; 6 Department of Emergency Medicine, University of Alberta, 790 University Terrace Building, 8303 – 112 Street, Edmonton, AB T6G 2T4 Canada; 7 Department of Family Practice, University of British Columbia, 3rd Floor David Strangway Building, 5950 University Boulevard, Vancouver, BC V6T 1Z3 Canada; 8 Emergency Strategic Clinical Network, Alberta Health Services, 14th Floor, North Tower, 10030 – 107 Street NW, Edmonton, AB T5J 3E4 Canada; 9 School of Population and Public Health and Centre for Health Services and Policy Research, University of British Columbia, 2206 East Mall, Vancouver, BC V6T1Z3 Canada

**Keywords:** Emergency medicine, Epidemiology, Health policy, Health services research, Frequent Users, High Service Users, Substance-Related Disorders

## Abstract

**Background:**

Substance use is common among people who visit emergency departments (EDs) frequently. We aimed to characterize subgroups within this cohort to better understand care needs/gaps, and generalizability of characteristics in three Canadian provinces.

**Methods:**

This was a retrospective cohort study (April 1^st^, 2013 to March 31^st^, 2016) of ED patients in Ontario, Alberta, and British Columbia (B.C.) We included patients ≥ 18 years with substance use-related healthcare contact during the study period and frequent ED visits, defined as those in the top 10% of ED utilization when all patients were ordered by annual ED visit number. We used linked administrative databases including ED visits and hospitalizations (all provinces); mental heath-related hospitalizations (Ontario and Alberta); and prescriptions, physician services, and mortality (B.C.). We compared to cohorts of people with (1) frequent ED visits and no substance use, and (2) non-frequent ED visits and substance use.

We employed cluster analysis to identify subgroups with distinct visit patterns and clinical characteristics during index year, April 1^st^, 2014 to March 31^st^, 2015.

**Results:**

In 2014/15, we identified 19,604, 7,706, and 9,404 people with frequent ED visits and substance use in Ontario, Alberta, and B.C (median 37–43 years; 60.9–63.0% male), whose ED visits and hospitalizations were higher than comparison groups.

In all provinces, cluster analyses identified subgroups with “extreme” and “moderate” frequent visits (median 13–19 versus 4–6 visits/year). “Extreme” versus “moderate” subgroups had more hospitalizations, mental health-related ED visits, general practitioner visits but less continuity with one provider, more commonly left against medical advice, and had higher 365-day mortality in B.C. (9.3% versus 6.6%; versus 10.4% among people with frequent ED visits and no substance use, and 4.3% among people with non-frequent ED visits and substance use). The most common ED diagnosis was acute alcohol intoxication in all subgroups.

**Conclusions:**

Subgroups of people with “extreme” (13–19 visits/year) and “moderate” (4–6 visits/year) frequent ED visits and substance use had similar utilization patterns and characteristics in Ontario, Alberta, and B.C., and the “extreme” subgroup had high mortality. Our findings suggest a need for improved evidence-based substance use disorder management, and strengthened continuity with primary and mental healthcare.

**Supplementary Information:**

The online version contains supplementary material available at 10.1186/s12873-022-00673-x.

## Background

Substance use disorders represent a substantial and growing burden of disease worldwide and in North America [1, 2]. In Canada, 21.6% of the total population (approximately 6 million people) meet criteria for substance use disorder at some point during their lifetime; alcohol use disorder is the most prevalent at 18.1% [3]. In the early stages of the COVID-19 pandemic, 28% of Canadians who used alcohol reported increasing their use, and 21% reported problematic use. However, only 24% of individuals with problematic substance use reported access to treatment [4]. People who use substances can have complex medical, social and mental health needs leading to emergency department (ED) visits. In North America, substance use accounts for one in 11 ED visits[5], and its healthcare burden is increasing over time [6, 7]. In particular, proportions of ED visits related to substance use disorders have increased since onset of COVID-19 [8, 9], indicating a growing need to recognize high risk substance use patterns in the ED to mitigate future harms.

ED visits may be the last point of healthcare system contact prior to premature death. In Alberta, Canada, 27% of individuals who died from opioid poisoning in 2017 had a past drug poisoning-related ED visit (19% had ≥ 3). Substance use often co-occurs with frequent ED utilization, which confers high mortality risk [10–12]. Previous analyses reported 9–12.5% 365-day mortality in Ontario and British Columbia (B.C.) [13, 14], Canada, compared to 3.5% among the general population with non-frequent ED visits [15]. There is mounting recognition and evidence that ED visits are key and often underrecognized opportunities to engage with people with substance use disorder and offer evidence-based supports. Motivational interview-based interventions in EDs have been shown to decrease alcohol consumption and alcohol-related injuries, and the American College of Emergency Physicians recommends ED-based screening and brief interventions for patients with harmful alcohol use [16]. Additionally, there is strong evidence to support ED-initiated buprenorphine/naloxone treatment for patients with opioid use disorder, which has been shown to decrease illicit opioid use and increase engagement with addictions care [17].

Previous literature has identified heterogeneous subgroups among people with frequent visits but has not explored subgroups among those specifically with substance use [14]. This characterization using multi-jurisdictional data is important to understand reasons underlying high healthcare utilization, risk profiles, and distinct needs to inform ED- and community-based interventions that may improve outcomes including mortality. The ED is a critical societal safety net, and people seeking ED care frequently can help us understand gaps in healthcare, social services, and other systems.

## Methods

### Objectives

The primary study objective was to characterize subgroups of people with frequent ED visits and substance use using cluster analysis in order to better understand care needs, gaps, and optimal resource allocation. To understand whether characteristics and gaps were generalizable in order to inform both regional and scalable multi-jurisdictional policies and approaches to improving care for these patients, a secondary objective was to provide descriptive data on subgroups in three Canadian provinces.

### Study design and setting

This was a retrospective cohort study using administrative data from April 1^st^, 2013 to March 31^st^, 2016 in the three Canadian provinces of Ontario, Alberta, and B.C.

Ontario is Canada’s most populous province, with a population of 15.0 million out of a total 38.5 million people in Canada. Its population has a median age of 40.7 years, it attracts 47.7% of all international immigrants to Canada, and 4.3% of the population identifies as Aboriginal [18, 19]. Alberta is the fourth most populous province, with 4.5 million residents. It has a median population age of 37.9 years and 6.1% of the population identifies as Aboriginal [18, 19]. B.C. is Canada’s third most populous province, with 5.3 million residents [18]. Its population has a median age of 42.3 years, it attracts 15.2% of all international immigrants to Canada, and 6.1% of its population identify as Aboriginal [18, 19]. Ontario accounts for the largest proportion of Canadian hospitals at 371 of 1265 (29%), compared to 175 in Alberta (14%) and 139 in B.C.(11%) [20].

### Data sources

#### Ontario and Alberta: Dynamic Cohort

We obtained Ontario and Alberta data from the Canadian Institute of Health Information (CIHI)’s Dynamic Cohort, a database that identifies patients in the top 10% of healthcare utilization [21]. For our study, we examined patients ≥ 18 years in the top 10% of ED visit utilization.

For our study database, CIHI linked patient-level Dynamic Cohort data to ED visits (National Ambulatory Care Reporting System [NACRS]) [22], hospitalizations (Discharge Abstract Database [DAD]) [23], and mental health hospitalizations (Hospital Mental Health Database [HMHDB]) [24] using personal health number. All Ontario and Alberta EDs comply with Level 3 NACRS reporting [25], which mandates capture of diagnoses using International Statistical Classification of Diseases and Related Health Problems 10th Revision (ICD-10-CA) [26]. The HMHDB collates information on mental health-related admissions from four sources, depending on jurisdiction: DAD, Hospital Morbidity Database (HMDB), Hospital Mental Health Survey (HMHS), and Ontario Mental Health Reporting System (OMHRS) [24, 27]. Because mental-health related admissions are variably recorded depending on Canadian jurisdiction, we included HMHDB data to allow for full characterization of mental health hospitalizations that would not have been captured in DAD. Additionally, HMHDB records a homelessness variable that is not captured in other databases and therefore allows unique insight into our characterized cohorts.

#### B.C.

We created a separate, linked database of people ≥ 18 years in B.C. who made ≥ 1 ED visit during the study timeframe using Population Data BC (PopData) data [28]. PopData undertakes database validation, quality assurance/control, and standardized linkage using personal health number, age, sex, and postal code [28, 29]. For our cohort, PopData linked patient-level data to ED visits (NACRS) and hospitalizations (DAD), as for Alberta and Ontario data, as well as to billed physician services (Medical Services Plan [MSP]) [22], prescriptions (PharmaNet)[25], and mortality (Vital Statistics), which we had access to only in.B.C [23].

B.C. EDs comply with Level 2 NACRS reporting, which as a quality assurance measure mandates that either ED Presenting Complaint or Discharge Diagnosis are completed [30]. For our descriptive and cluster analyses, we focused on ED discharge diagnoses.

### Study cohort

We first identified patients who visited EDs frequently from April 1^st^, 2014 to March 31^st^, 2015, defined as patients ≥ 18 years in the top 10% of ED utilization when all patients were ordered by ED visits per fiscal year; each annual cohort represented the top 10% in that specific year. CIHI identified this cohort a priori in Ontario and Alberta within the Dynamic Cohort. We applied the 10% threshold definition to our B.C. data.

Among this cohort, we then identified patients who had made healthcare contact for substance use, defined as ≥ 1 substance use-related ED visit (NACRS), hospitalization (DAD), mental health admission (HMHDB [Ontario and Alberta]), or billed physician services (MSP [B.C.]) during the study period.

### Comparison cohorts

To provide context, we also characterized patients ≥ 18 years in the top 10% of ED utilization with no healthcare contact for substance use, and patients ≥ 18 years in the bottom 90% of ED utilization (non-frequent ED visits) who did make healthcare contact for substance use. We characterized the demographics, healthcare utilization, and mortality of these comparison cohorts using the same a priori list of variables.

### Study variables and definitions

#### Substance use

We used CIHI's definition of substance use-related presentations based on ICD-10-CA and ICD-9 codes (Supplementary Table 1) [31], which fall into four categories related to alcohol, opioids, stimulants, and other substances. We assessed for substance use-related healthcare encounters at any time during the study timeframe.

#### Index year and index visit

For our cluster analysis, we defined April 1^st^, 2014 to March 31^st^, 2015 as the index year, and each patient’s index visit as their last visit therein. We used a 365-day pre-index period to examine baseline variables for our cluster algorithm. We assessed mortality in the 365 days post-index visit in B.C.

#### Demographics and healthcare utilization

We developed an a priori list of variables based on our team’s clinical expertise, to characterize demographics, healthcare utilization, and outcomes for our population of interest. All variables and their sources are summarized in Supplementary Table 2. We characterized demographics and ED visits using NACRS, hospitalizations using DAD, HMHDB-reported mental health admissions (Ontario and Alberta), and for B.C. patients, billed physician services using MSP, prescriptions using PharmaNet, and mortality using Vital Statistics.

#### Diagnostic categories

In NACRS and DAD, we summarized ICD-10-CA diagnoses, diagnostic categories using the 22 ICD-10-CA chapters, and substance-related diagnoses using sub-chapters within Chapter F10 (Mental and behavioral disorders due to psychoactive substance use).

The HMHDB reports diagnoses within mental health categories using ICD-10-CA (DAD), and the Diagnostic and Statistical Manual of Mental Disorders (OMHRS and HMHS) [24].

#### ED visit acuity

NACRS classifies ED visit acuity using the Canadian Triage and Acuity Scale (CTAS), a national five-level system to prioritize ED patient care [24]. CTAS has good inter-rater reliability, and predictive validity for admission and mortality [32, 33].

#### Majority source of care

For B.C. patients, we identified primary care physicians and visits using MSP practitioner codes. We created a binary Majority Source of Care variable assessed in the pre-index period [34]. This standard for primary care continuity identifies whether, among patients with ≥ 3 annual general practitioner services, ≥ 50% were provided by one practitioner.

#### Prescriptions

For B.C. patients, we identified prescriptions in Pharmanet using drug and product identification numbers [35, 36].

#### Housing status

Homelessness was documented in HMHDB. Though unvalidated, it is derived from mandatory reporting fields: “postal code” within DAD (Ontario and Alberta) and “Usual Residential Status” in OMHRS (Ontario only) [37].

#### Regularity index of ED visits

We calculated a regularity index (ranging from 0 to 1; closer to 1 indicating greater regularity) to characterize spacing between patients’ ED visits over the 365-day pre-index visit period: (1)/_1+visit variance_, with variance based on days between visits [38]. To illustrate, a person with 12 equally spaced monthly visits would have an index of 1, whereas a person with 12 randomly dispersed annual visits would have an index close to 0.

#### Charlson comorbidity index

We calculated a Charlson Comorbidity Index for each individual by assessing patients’ status on 17 comorbidities using NACRS primary ED diagnoses. This index has predictive validity for mortality and has been validated using ED data [39, 40].

#### Data analyses

##### Descriptive statistics

We described demographics, healthcare utilization, and mortality for people with frequent ED use and substance use, people with frequent ED visits and no substance use, and people with non-frequent ED visits and substance use in each province from April 1^st^, 2014 to March 31^st^, 2015. Since our characterizations were exploratory in nature, we summarized characteristics descriptively without undertaking testing of statistical significance.

##### Cluster analyses

We employed cluster analysis, commonly used in similar applications [41], to identify subgroups within our cohort with frequent ED visits and substance use during index year April 1^st^, 2014 to March 31^st^, 2015. This method organizes data by optimizing within-subgroup similarities and between-subgroup differences [42, 43].

For our clustering algorithm, we included variables describing visit patterns and clinical characteristics. As is a common approach, our previous analyses and clinical experience informed variable inclusion: [14, 44]

1. Age at index visit;
2. ED visit number;
3. Charlson comorbidity index;
4. Number of months that the patient visited an ED;
5. Regularity index;

Number of ICD-10-CA ED discharge diagnoses pertaining to:

6. alcohol;
7. stimulants and psychoactive drugs;
8. opioids;
9. other substances;
10. number of distinct substances.

We identified clusters using a *Kmeans *algorithm, then used elbow plots in conjunction with pseudo-F tests to determine that two clusters optimally described our data (Supplementary Tables 3,4,5; Supplementary Figs. 1,2, 3) [43, 45].

We performed all analyses using R (R Development Core Team, 2011).

### Ethics approval

The University of British Columbia (UBC) Clinical Research Ethics Board approved our study (H18-00,287 & H18-00,708). We received approvals from CIHI and PopData to access the patient data used in our research. Since we analyzed aggregate provincial administrative data, the UBC Clinical Research Ethics Board deemed it unnecessary to obtain individual informed consent from all participants in the study.

## Results

In 2014/15, our index year, we identified 19,604 people with frequent ED visits and substance use in Ontario, 7,706 in Alberta, and 9,404 in B.C. These patients made a median 5–6 ED visits/year and 42.3–65.5% had at least one admission per year (Table 1). They accounted for 4.2%, 7.4%, and 9.7% of all people with frequent ED use in each province. Patients were young (median 37–43 years) and majority male (60.9–63.0%). In comparison, people with frequent ED visits and no healthcare contact for substance use made 3–5 median ED visits/year, a smaller proportion (32.6–44.3%) were hospitalized at least once per year, median age was older (46–56 years), and a smaller proportion (54.9–56.5%) were male. People with non-frequent ED visits (bottom 90%) and contact for substance use made one ED visit/year, and fewer (25.8–41.1%) had at least one admission per year. Similarly to those with frequent ED visits and substance use, median age was 36–43 years, and a majority (62.6%-64.0%) were male.

**Table 1:** Characteristics of people with (1) frequent ED visits (top 10%) and substance use; (2) frequent ED visits (top 10%) and no substance use; and (3) non-frequent ED visits (bottom 90%) and substance use in Ontario, Alberta, and B.C. (April 1^st^, 2014 to March 31^st^, 2015)

Characteristics	Ontario	Alberta	B.C
**Number of patients with at least one ED visit**	**2,572,057**	**841,346**	**643,000**
**Number of people with frequent ED visits (top 10%)**	**467,223**	**106,381**	**97,120**
**(1) Number of people with frequent ED visits (top 10%) and substance use**	**19,604**	**7,706**	**9,404**
**Sex, n (%)**			
Male	12,115 (61.8)	4,695 (60.9)	5,922 (63.0)
Female	7,489 (38.2)	3,011 (39.1)	3,482 (37.0)
**Age at year end (years)**			
Median (IQR)	40 (27–53)	37 (27–50)	43 (30–55)
**Urban/rural residence, n (%)**			
Rural	2,760 (14.1)	2,032 (26.4)	470 (5.0)
Urban	15,985 (81.5)	5,001 (64.9)	8,862 (94.2)
NA	859 (4.4)	673 (8.7)	72 (0.8)
**Neighborhood income quintile, n (%)**			
1st Quintile	5,094 (26.0)	2,822 (36.6)	3,677 (39.1)
2nd Quintile	3,993 (20.4)	1,045 (13.6)	1,811 (19.3)
3rd Quintile	3,326 (17.0)	1,469 (19.1)	1,478 (15.7)
4th Quintile	3,045 (15.5)	781 (10.1)	1,195 (12.7)
5th Quintile	3,131 (16.0)	708 (9.2)	925 (9.8)
Unknown	156 (0.8)	208 (2.7)	245 (2.6)
**Total number of ED visits**	**153,763**	**71,835**	**63,809**
**Number of ED visits per patient**			
Median (IQR)	5 (3–8)	6 (5–10)	5 (3–7)
**Total number of admissions**	17,547	10,555	15,245
**Number of patients with at least one admission, n (%)**	8,292 (42.3)	4,575 (59.4)	6,161 (65.5)
**(2) Number of people with frequent ED visits (top 10%) and no substance use**	**447,619 (100)**	**98,675 (100)**	**87,716 (100)**
**Sex, n (%)**			
Male	194,507 (43.5)	43,433 (44.0)	39,539 (45.1)
Female	253,110 (56.5)	55,242 (56.0)	48,157 (54.9)
Unknown	2 (0.0)	0 (0.0)	20 (0.0)
**Age at year end (years)**			
Median (IQR)	52 (33–70)	46 (30–64)	56 (36–74)
**Urban/rural residence, n (%)**			
Rural	94,310 (21.1)	34,519 (35.0)	3,505 (4.0)
Urban	352,038 (78.6)	63,369 (64.2)	82,323 (93.9)
NA	1,271 (0.3)	787 (0.8)	1,888 (2.2)
**Neighborhood income quintile, n (%)**			
1st Quintile	100,949 (22.6)	38,638 (39.2)	22,801 (26.0)
2nd Quintile	95,055 (21.2)	13,456 (13.6)	18,591 (21.2)
3rd Quintile	78,814 (17.6)	19,859 (20.1)	16,413 (18.7)
4th Quintile	84,536 (18.9)	13,020 (13.2)	15,310 (17.5)
5th Quintile	85,691 (19.1)	10,488 (10.6)	13,175 (15.0)
Unknown	2,574 (0.6)	3,214 (3.3)	1,426 (1.6)
**Total number of ED visits**	**1,996,495**	**632,736**	**379,037**
**Number of ED visits per patient**			
Median (IQR)	4 (3–5)	5 (4–7)	3 (3–5)
**Total number of admissions**	267,415	63,082	77,379
**Number of patients with at least one admission**	146,047 (32.6)	33,454 (33.9)	38,836 (44.3)
**(3) Number of people with non-frequent ED visits (bottom 90%) and substance use**	**6,088 (100)**	**2,742 (100)**	**12,967 (100)**
**Sex, n (%)**			
Male	3,895 (64.0)	1,717 (62.6)	8164 (63.0)
Female	2,192 (36.0)	1,025 (37.4)	4802 (37.0)
Unknown	1 (0.0)	0 (0.0)	< 5 (0.0)
**Age at year end (years)**			
Median (IQR)	37 (23–53)	36 (24–51)	43 (29–56)
**Urban/rural residence, n (%)**			
Rural	755 (12.4)	456 (16.6)	836 (6.4)
Urban	5,165 (84.8)	2,203 (80.3)	11,060 (85.3)
NA	168 (2.8)	83 (3.0)	1,071 (8.3)
**Neighborhood income quintile, n (%)**			
1st Quintile	1,349 (22.2)	802 (29.2)	3,915 (30.2)
2nd Quintile	1,297 (21.3)	466 (17.0)	2,623 (20.2)
3rd Quintile	1,047 (17.2)	605 (22.1)	2,229 (17.2)
4th Quintile	1,083 (17.8)	374 (13.6)	2,036 (15.7)
5th Quintile	1,075 (17.7)	351 (12.8)	1,744 (13.4)
Unknown	237 (3.9)	144 (5.3)	420 (3)
**Total number of ED visits**	**9,903**	**15,328**	**17,643**
**Number of ED visits per patient**			
Median (IQR)	1 (1–2)	1 (1–4)	1 (1–2)
**Total number of admissions**	1,844	1,521	8,050
**Number of patients with at least one admission**	1,568 (25.8)	1,117 (40.7)	5,323 (41.1)

### Subgroups identified by cluster analysis

Our cluster analyses yielded similar characterizations in Ontario, Alberta, and B.C. In all three provinces, we identified two subgroups distinguished largely by ED visit number: a subgroup with extremely frequent visits (“extreme”; median visits/year in Ontario: 19; Alberta: 19; B.C.: 13) and a subgroup with moderately frequent visits (“moderate”; median visits/year in Ontario: 5; Alberta: 6; B.C.: 4) (Table 2 and Supplementary Table 6).

**Table 2:** Demographic and ED utilization characteristics of subgroups of people with frequent ED visits (top 10%) and substance use in Ontario, Alberta, and B.C. (April 1^st^, 2014 to March 31^st^, 2015)

Characteristics	Ontario		Alberta		B.C	
	**Extreme (*N* = 2,669)**	**Moderate (*N* = 16,921)**	**Extreme (*N* = 1,468)**	**Moderate (*N* = 6,220)**	**Extreme (*N* = 1,690)**	**Moderate (*N* = 7,713)**
***Subgroup Characteristics (Selected Clustering Variables)***						
** Age (years)**						
Median (IQR)	36 (27–49)	40 (27–54)	38 (29–49)	37 (27–50)	45 (33–53)	44 (31–56)
** Total number of ED visits**						
Median (IQR)	19 (13–28)	5 (4–8)	19 (15–27)	6 (5–9)	13 (10–20)	4 (3–6)
** Number of alcohol-related ED visits**						
Median (IQR)	1 (0–7)	0 (0–1)	2 (0–7)	1 (0–2)	1 (0–4)	0 (0–1)
** Weighted Charlson Comorbidity Index**						
Median (IQR)	0 (0–0)	0 (0–0)	0 (0–0)	0 (0–0)	0 (0–0)	0 (0–0)
***Patient Characteristics (NACRS)***						
** Number of patients**	**2,669 (13.6)**	**16,921 (86.4)**	**1,468 (19.1)**	**6,220 (80.9)**	**1,690 (18.0)**	**7,713 (82.0)**
** Sex, n (%)**						
Male	1,675 (62.8)	10,434 (61.7)	888 (60.5)	3,791 (60.9)	1,148 (67.9)	4,774 (61.9)
Female	994 (37.2)	6,487 (38.3)	580 (39.5)	2,429 (39.1)	542 (32.1)	2,939 (38.1)
NA	0 (0.0)	0 (0.0)	0 (0.0)	0 (0.0)	0 (0.0)	0 (0.0)
** Neighborhood income quintile, n (%)**						
1^st^ Quintile	704 (26.4)	4,391 (26.0)	543 (37.0)	2,272 (36.5)	787 (46.6)	2,880 (37.3)
2^nd^ Quintile	518 (19.4)	3,476 (20.5)	188 (12.8)	850 (13.7)	285 (16.9)	1,525 (19.8)
3^rd^ Quintile	371 (13.9)	2,944 (17.4)	228 (15.5)	1,242 (20.0)	248 (14.7)	1,238 (16.1)
4^th^ Quintile	368 (13.8)	2,673 (15.8)	119 (8.1)	662 (10.6)	172 (10.2)	1,024 (13.3)
5^th^ Quintile	398 (14.9)	2,731 (16.1)	121 (8.2)	584 (9.4)	134 (7.9)	795 (10.3)
Unknown	47 (1.8)	156 (0.9)	22 (2.2)	157 (2.5)	56 (3.3)	188 (2.4)
NA	263 (9.9)	550 (3.3)	237 (16.1)	453 (7.3)	8 (0.5)	63 (0.8)
** Urban/rural residence, n (%)**						
Urban	2,147 (80.4)	13,832 (81.7)	861 (58.7)	4,091 (65.8)	1,637 (96.9)	7,224 (93.7)
Rural	259 (9.7)	2,539 (15.0)	370 (25.2)	1,676 (26.9)	42 (2.5)	428 (5.5)
NA	263 (9.9)	550 (3.3)	237 (16.1)	453 (7.3)	11 (0.7)	61 (0.8)
***ED Visit Characteristics (NACRS)***						
** Number of ED visits (for any reason)**	**66,655**	**102,807**	**34,400**	**44,193**	**28,893**	**39,181**
** Arrival by ambulance among ED visits, n (%)**						
Ground and/or air ambulance	29,003 (43.5)	35,630 (34.7)	12,243 (35.6)	13,637 (30.9)	14,267 (49.4)	15,192 (38.8)
No ambulance	37,652 (56.5)	67,177 (65.3)	22,157 (64.4)	30,556 (69.1)	14,626 (50.6)	23,989 (61.2)
** Disposition group among ED visits, n (%)**						
Discharged	49,084 (73.6)	70,767 (68.8)	25,250 (73.4)	31,602 (71.5)	23,058 (79.8)	28,729 (73.3)
Left AMA	8,099 (12.2)	8,351 (8.1)	5,052 (14.7)	4,426 (10.0)	635 (2.2)	558 (1.4)
Admitted/Transferred	9,462 (14.2)	23,642 (23)	4,089 (11.9)	8,155 (18.5)	5,196 (18.0)	9,882 (25.2)
Died	10 (0.0)	47 (0.0)	9 (0.0)	10 (0.0)	< 5 (0.0)	12 (0.0)
NA	0 (0.0)	0 (0.0)	0 (0.0)	0 (0.0)	0 (0.0)	0 (0.0)
** Substance-related diagnostic categories among ED visits, n (%)**						
Alcohol	14,937 (22.4)	16,305 (15.9)	7,562 (22.0)	7,140 (16.2)	5,623 (19.5)	4,642 (11.8)
Opioids	1,343 (2.0)	2,879 (2.8)	538 (1.6)	953 (2.2)	475 (1.6)	481 (1.2)
Sedative/Hypnotics	668 (1.0)	1,579 (1.5)	300 (0.9)	577 (1.3)	< 5 (0.0)	< 5 (0.0)
Stimulants, Cocaine, Psychoactive and Hallucinogens	4,241 (6.4)	4,584 (4.5)	1,596 (4.6)	2,086 (4.7)	1,341 (4.6)	1,201 (3.1)
Other Substances	248 (0.4)	749 (0.7)	67 (0.2)	166 (0.4)	67 (0.2)	35 (0.1)
Non substance-related visit	45,218 (67.8)	76,711 (74.6)	24,337 (70.7)	33,271 (75.3)	21,385 (74)	22,858 (58.3)
NA	0 (0.0)	0 (0.0)	0 (0.0)	0 (0.0)	5,004 (17.3)	9,962 (25.4)
** Top 3 ED diagnoses (ICD-10-CA)**						
1	Mental and behavioural disorders due to use of alcohol, acute intoxication (F100): 7,409 (11.1)	Mental and behavioural disorders due to use of alcohol, acute intoxication (F100): 4,648 (4.5)	Mental and behavioural disorders due to use of alcohol, acute intoxication (F100): 3,173 (9.2)	Mental and behavioural disorders due to use of alcohol, acute intoxication (F100): 1,968 (4.5)	Mental dis alcohol intoxicat (F100): 2,995 (10.4)	Mental dis alcohol intoxicat (F100): 2,144 (5.5)
2	Mental and behavioural disorders due to use of alcohol, harmful use (F101): 3,592 (5.4)	Mental and behavioural disorders due to use of alcohol, harmful use (F101): 4,037 (3.9)	Mental and behavioural disorders due to use of alcohol, harmful use (F101): 1,387 (4.0)	Mental and behavioural disorders due to use of alcohol, withdrawal state (F103): 1,546 (3.5)	Toxic effects ethanol (T510): 1,799 (6.2)	Toxic effects ethanol (T510): 1,734 (4.4)
3	Mental and behavioural disorders due to use of alcohol, withdrawal state (F103): 2,133 (3.2)	Mental and behavioural disorders due to use of alcohol, withdrawal state (F103): 3,771 (3.7)	Mental and behavioural disorders due to use of alcohol, withdrawal state (F103): 1,290 (3.8)	Mental and behavioural disorders due to use of alcohol, harmful use (F101): 1,455 (3.3)	Abdominal pain (R104): 1.231 (4.3)	Abdominal pain (R104): 1120 (2.9)

We summarize subgroups’ demographic, clinical, and healthcare utilization characteristics in Tables 2 and 3, and Supplementary Table 6.

**Table 3:** Hospitalization and healthcare utilization characteristics of subgroups of people with frequent ED visits (top 10%) and substance use in Ontario, Alberta, and B.C. (April 1^st^, 2014 to March 31^st^, 2015)

Characteristics	Ontario		Alberta		B.C	
***Hospitalization Characteristics (DAD)***						
	**Extreme (*N* = 2,669)**	**Moderate (*N* = 16,921)**	**Extreme (*N* = 1,468)**	**Moderate (*N* = 6,220)**	**Extreme (*N* = 1,690)**	**Moderate (*N* = 7,713)**
**Number of patients with at least one admission,** n (%)	**1,444 (54.1)**	**6,873 (40.6)**	**1,068 (72.8)**	**3,425 (55.1)**	**1,374 (81.3)**	**4,756 (61.7)**
**Number of admissions per patient**						
Median (IQR)	2 (1–4)	1 (1–2)	2 (1–5)	2 (1–3)	3 (2–5)	2 (1–3)
**Number of admissions (for any reason)**	**4,498**	**13,648**	**3,647**	**6,981**	**5,499**	**10,446**
**Total length of stay among admissions (days)**						
Median (IQR)	2 (1–5)	3 (2–7)	3 (2–7)	4 (2–9)	3 (1–6)	3 (1–8)
**Disposition group among admissions, n (%)**						
Transferred	656 (14.6)	2,157 (15.8)	279 (7.7)	699 (10)	493 (9.0)	1,052 (10.1)
Discharged	3,203 (71.2)	10,282 (75.3)	2,756 (75.6)	5,547 (79.5)	4,163 (75.7)	8,302 (79.5)
Signed out (against medical advice)	621 (13.8)	927 (6.8)	586 (16.1)	639 (9.2)	807 (14.7)	961 (9.2)
Died	18 (0.4)	280 (2.1)	22 (0.6)	84 (1.2)	25 (0.5)	99 (0.9)
Did not return from pass	0 (0.0)	2 (0.0)	4 (0.1)	12 (0.2)	11 (0.2)	32 (0.3)
NA	0 (0.0)	0 (0.0)	0 (0.0)	0 (0.0)	0 (0.0)	0 (0.0)
**Substance-related diagnostic categories among admissions, n (%)**						
Alcohol	1,108 (24.6)	3,891 (28.5)	1,084 (29.7)	1,951 (27.9)	1,415 (25.7)	2,177 (20.8)
Other substances	417 (9.3)	1,340 (9.8)	427 (11.7)	844 (12.1)	901 (16.4)	1,841 (17.6)
Non substance-related admission	2,973 (66.1)	8,417 (61.7)	2,136 (58.6)	4,186 (60)	3,183 (57.9)	6,428 (61.5)
**Top 3 primary diagnosis among admissions, n (%)**						
1	Mental and behavioural disorders due to use of alcohol, withdrawal state (F103): 477 (10.6)	Mental and behavioural disorders due to use of alcohol, withdrawal state (F103): 1,029 (7.5)	Mental and behavioural disorders due to use of alcohol, withdrawal state (F103): 462 (12.7)	Mental and behavioural disorders due to use of alcohol, withdrawal state (F103): 558 (8.0)	Mental and behavioural disorders due to use of alcohol, withdrawal state (F103): 504 (9.2)	Mental and behavioural disorders due to use of alcohol, withdrawal state (F103): 613 (5.9)
2	Mental and behavioural disorders due to use of alcohol, acute intoxication (F100): 164 (3.6)	Alcohol-induced acute pancreatitis (K852): 648 (4.7)	Mental and behavioural disorders due to use of alcohol, dependence syndrome (F102): 109 (3.0)	Alcohol-induced acute pancreatitis (K852): 230 (3.3)	Mental and behavioural disorders due to use of alcohol, acute intoxication (F100): 259 (4.7)	Mental and behavioural disorders due to use of alcohol, acute intoxication (F100): 331 (3.2)
3	Poisoning by benzodiazepines (T424): 99 (2.2)	Alcoholic cirrhosis of liver (K703): 588 (4.3)	Mental and behavioural disorders due to use of alcohol, acute intoxication (F100): 90 (2.5)	Mental and behavioural disorders due to use of alcohol, dependence syndrome (F102): 226 (3.2)	Mental and behavioural disorders due to use of other stimulants including caffeine, psychotic disorder (F155): 210 (3.8)	Mental and behavioural disorders due to use of other stimulants including caffeine, psychotic disorder (F155): 306 (2.9)
***Mental Health Admission Characteristics (HMHDB)***						
	**Extreme (*N* = 2,669)**	**Moderate (*N* = 16,921)**	**Extreme (*N* = 1,468)**	**Moderate (*N* = 6,220)**	**Extreme (*N* = 1,690)**	**Moderate (*N* = 7,713)**
**Number of patients with at least one mental health admission,** n (%)	**1,405 (52.6)**	**6,054 (35.8)**	**695 (47.3)**	**1,849 (29.7)**	NA	NA
**Number of mental health admissions per patient**						
Median (IQR)	2 (1–4)	1 (1–2)	2 (1–3)	1 (1–2)	NA	NA
**Homelessness among patients with at least one admission, n (%)**						
No	1,197 (85.2)	5,770 (95.3)	553 (79.6)	1,720 (93.0)	NA	NA
Yes	208 (14.8)	284 (4.7)	142 (20.4)	129 (7.0)	NA	NA
**Number of mental health admissions (for any reason)**	**4,294**	**10,229**	**1,605**	**2,876**	NA	NA
Discharge disposition among mental health admissions, n (%)*						
Discharged Home	3,191 (74.3)	8,553 (83.6)	1,200 (74.8)	2,305 (80.1)	NA	NA
Transferred	286 (6.7)	757 (7.4)	106 (6.6)	248 (8.6)	NA	NA
Died	0 (0.0)	4 (0.0)	0 (0.0)	3 (0.1)	NA	NA
Sign-out	276 (6.4)	347 (3.4)	299 (18.6)	320 (11.1)	NA	NA
Other	541 (12.6)	568 (5.6)	0 (0.0)	0 (0.0)	NA	NA
**Diagnostic categories among mental health admissions, n (%)**						
Organic Disorders	31 (0.7)	164 (1.6)	9 (0.6)	56 (1.9)	NA	NA
Substance Related Disorders	2,220 (51.7)	5,517 (53.9)	1162 (72.4)	1,891 (65.8)	NA	NA
Schizophrenic and Psychotic Disorders	665 (15.5)	1,340 (13.1)	110 (6.9)	276 (9.6)	NA	NA
Mood Disorders	679 (15.8)	2,123 (20.8)	125 (7.8)	305 (10.6)	NA	NA
Anxiety Disorders	108 (2.5)	258 (2.5)	17 (1.1)	72 (2.5)	NA	NA
Personality Disorders	392 (9.1)	316 (3.1)	78 (4.9)	65 (2.3)	NA	NA
Other Mental Health Disorders	185 (4.3)	468 (4.6)	103 (6.4)	207 (7.2)	NA	NA
Unknown Disorders	1 (0.0)	6 (0.1)	0 (0.0)	0 (0.0)	NA	NA
Non-Mental Health Disorder	13 (0.3)	37 (0.4)	1 (0.1)	4 (0.1)	NA	NA
***Other Healthcare Utilization Characteristics (MSP and Pharmanet)***						
	**Extreme (*N* = 2,669)**	**Moderate (*N* = 16,921)**	**Extreme (*N* = 1,468)**	**Moderate (*N* = 6,220)**	**Extreme (*N* = 1,690)**	**Moderate (*N* = 7,713)**
**MSP Long-term care, n (%)**						
No	NA	NA	NA	NA	1,655 (97.9)	7,278 (94.4)
Yes	NA	NA	NA	NA	13 (0.8)	63 (0.8)
NA	NA	NA	NA	NA	22 (1.3)	372 (4.8)
**MSP number of general practitioner visits**						
Median (IQR)	NA	NA	NA	NA	53 (26–97)	28 (13–55)
**MSP number of individual general practitioners**						
Median (IQR)	NA	NA	NA	NA	14 (7–30)	12 (6–25)
**MSP Majority source of care, n (%)**						
Yes	NA	NA	NA	NA	389 (23.0)	3,010 (39.0)
No	NA	NA	NA	NA	1,279 (75.7)	4,331 (56.2)
0 GP visit	NA	NA	NA	NA	22 (1.3)	372 (4.8)
**Number of prescription medications based on DINPIN**						
Median (IQR)	NA	NA	NA	NA	26 (16–39)	19 (11–29)

#### Demographics

In B.C., “extreme” versus “moderate” subgroups were disproportionately represented in the lowest neighborhood quintile, although this was not seen in Ontario and Alberta. Among those with a HMHDB-reported admission, “extreme” versus “moderate” subgroups had more documented homelessness (14.8% versus 4.7% in Ontario; and 20.4% versus 7.0% in Alberta).

#### Clinical characteristics

“Extreme” versus “moderate” subgroups had a higher proportion of mental health-related ED visits (41.7% versus 32.7% in Ontario; 31.5% versus 27.3% in Alberta; and 24.7% versus 18.8% in B.C.), and mental health-related hospitalizations documented in both DAD (24.6% versus 19.7% in Ontario; 43.8% versus 41% in Alberta; and 51.1% versus 47.1% in B.C.) and HMHDB (52.6% versus 35.8% in Ontario; 47.3% versus 29.7% in Alberta). The “moderate” subgroup had comparatively more digestive system-related ED visits and hospitalizations, among which pancreatitis, alcoholic cirrhosis, and alcoholic gastritis were common. Both subgroups demonstrated low Charlson comorbidity indices (median 0) in all provinces, indicating a lack of chronic medical comorbidities among these patients. Among ED visits, alcohol was the most prevalent substance and accounted for proportionally more visits within the “extreme” subgroup. Among all subgroups, the most common discharge diagnoses were alcohol intoxication (ED visits) and alcohol withdrawal (hospitalizations).

#### Healthcare utilization

In all provinces, a greater proportion of ED visits made by patients in the “extreme” versus “moderate” subgroup arrived by ambulance (43.5% versus 34.7% in Ontario; 35.6% versus 30.9% in Alberta; and 49.4% versus 38.8% in B.C.). Furthermore, greater proportions of patients in the “extreme” versus “moderate” subgroups were hospitalized at least once in the index year (54.1% versus 40.6% in Ontario; 72.8% versus 55.1% in Alberta; and 81.3% versus 61.7% in B.C.). In B.C., the “extreme” subgroup had more prescriptions, made more general practitioner visits, and saw more individual general practitioners, however, a smaller proportion had one physician as their majority source of care (23.0% versus 39.0%).

#### Visit dispositions

A greater proportion of patients in the “extreme” versus “moderate” subgroups were discharged or left against medical advice but a smaller proportion were admitted following individual ED visits.

Additionally, a greater proportion of patients in the “extreme” versus “moderate” subgroups left against medical advice during a hospitalization.

#### Subgroup mortality

In B.C., 7.1% (*n* = 667) of people with frequent ED visits and substance use died within 365 days of index visit in 2014/2015: 9.3% of people in the “extreme” versus 6.6% in the “moderate” subgroup (Table 4). In comparison, people with frequent ED visits and no substance use had a 365-day mortality of 10.4% and people with non-frequent ED visits and substance use had a 365-day mortality of 4.3% during the same timeframe (Table 4). Deceased patients in “extreme” subgroup had a median age of 52 (IQR: 43–61), while those in the “moderate” subgroup had a median age of 59 (IQR: 46–71). In the “extreme” subgroup, alcoholic cirrhosis and alcoholic liver disease accounted for 16.4% of deaths. In the “moderate” subgroup, alcoholic liver disease and alcoholic hepatic failure accounted for 8.4% of deaths. Chronic hepatitis C accounted for 6.3% and 9.8% of deaths in “extreme” and “moderate” subgroups, respectively. In comparison, people with frequent ED visits and no substance use most commonly died of lung malignancy (9.0%) and 7.6% died of atherosclerotic heart disease or myocardial infarction. Among people with non-frequent ED visits and substance use, alcoholic cirrhosis and hepatic failure accounted for 18.9% of all deaths.

**Table 4:** Characteristics of (1) Extreme and moderate subgroups of people with frequent ED visits (top 10%) and substance use; (2) People with frequent ED visits (top 10%) and no substance use; and (3) People with non-frequent ED visits (bottom 90%) and substance use who died within 365 days of last ED visit from April 1^st^, 2014 to March 31^st^, 2015 in B.C

	**Extreme subgroup of people with frequent ED visits and substance use (*N* = 1,690)**	**Moderate subgroup of people with frequent ED visits and substance use (*N* = 7,713)**	**People with frequent ED visits (top 10%) and no substance use (*N* = 87,716)**	**People with non-frequent ED visits (bottom 90%) and substance use (*N* = 12,967)**
**Number of deaths**	**158 (9.3)**	**509 (6.6)**	**9,141 (10.4)**	**561 (4.3)**
**Sex among deaths, n (%)**				
Male	113 (71.5)	347 (68.2)	4,751 (52.0)	375 (66.8)
Female	45 (28.5)	162 (31.8)	4,383 (47.9)	186 (33.2)
Unknown	0 (0.0)	0 (0.0)	7 (0.1)	0 (0.0)
**Age among deaths**				
Median (IQR)	52 (43–61)	59 (46–71)	80 (68–88)	61 (51–72)
**Neighborhood income quintile among deaths, n (%)**				
1^st^ Quintile	75 (47.5)	200 (39.3)	2,336 (25.6)	183 (32.6)
2^nd^ Quintile	25 (15.8)	101 (19.8)	1,962 (21.5)	100 (17.8)
3^rd^ Quintile	24 (15.2)	92 (18.1)	1,732 (18.9)	105 (18.7)
4^th^ Quintile	10 (6.3)	59 (11.6)	1,593 (17.4)	86 (15.3)
5^th^ Quintile	12 (7.6)	46 (9)	1,428 (15.6)	71 (12.7)
Unknown	8 (5.1)	8 (1.6)	79 (0.1)	< 5 (0.4)
NA	< 5 (2.5)	< 5 (0.6)	11 (0.9)	14 (2.5)
**Urban/rural among deaths, n (%)**				
Urban	152 (96.2)	463 (91.0)	8,647 (94.6)	480 (85.6)
Rural	< 5 (1.3)	43 (8.4)	404 (4.4)	59 (10.5)
NA	< 5 (2.5)	< 5 (0.6)	90 (1.0)	22 (3.9)
**MSP long-term care among deaths, n (%)**				
No	153 (96.8)	485 (95.3)	8,164 (89.3)	485 (86.5)
Yes	5 (3.2)	24 (4.7)	971 (10.6)	34 (6.1)
NA	0 (0.0)	11 (2.2)	6 (0.1)	42 (7.5)
**MSP majority source of care among deaths, n (%)**				
Yes	36 (22.8)	203 (39.9)	5,034 (55.1)	335 (59.7)
No	122 (77.2)	295 (58.0)	4,062 (44.4)	148 (26.4)
0 GP visit	0 (0.0)	0 (0.0)	39 (0.4)	36 (6.3)
NA	0 (0.0)	11 (2.2)	6 (0.1)	42 (7.5)
**MSP number of general practitioner visits among deaths**				
Median (IQR)	67 (35–118)	54 (26–96)	49 (28–83)	14 (6–36)
Range	3–344	2–340	1–607	1–238
**MSP number of individual general practitioner visits among deaths**				
Median (IQR)	18 (8–34)	22 (11–39)	24 (14–40)	8 (4–18)
Range	1–231	1–223	1–336	1–180
**Top 5 cause of deaths**				
1	Other ill-defined and unspecified causes of mortality (R99): 31 (19.6)	Other ill-defined and unspecified causes of mortality (R99): 82 (16.1)	Malignant neoplasm of right bronchus or lung unspecified (C349): 827 (9.0)	Other ill-defined and unspecified causes of mortality (R99): 77 (13.7)
2	Alcoholic cirrhosis of liver (K703): 19 (12.0)	Chronic viral hepatitis C (B182): 50 (9.8)	Atherosclerotic heart disease of native coronary artery (I251): 398 (4.4)	Alcoholic cirrhosis of liver (K703): 69 (12.3)
3	Chronic viral hepatitis C (B182): 10 (6.3)	Accidental poisoning by and exposure to other and unspecified drugs, medicaments and biological substances (X44): 24 (4.7)	Unspecified dementia (F03): 299 (3.3)	Alcoholic hepatic failure (K704): 37 (6.6)
4	Accidental poisoning by and exposure to other and unspecified drugs, medicaments and biological substances (X44): 9 (5.7)	Alcoholic liver disease unspecified (K709): 22 (4.3)	Acute myocardial infarction, unspecified (I219): 292 (3.2)	Accidental poisoning by and exposure to other and unspecified drugs, medicaments and biological substances (X44): 25 (4.5)
5	Alcoholic liver disease unspecified (K709): 7 (4.4)	Alcoholic hepatic failure (K704): 21 (4.1)	Chronic obstructive pulmonary disease, unspecified (J449): 269 (2.9)	Malignant neoplasm of right bronchus or lung unspecified (C349): 20 (3.6)

## Discussion

### Interpretation and findings

Our cluster analysis identified two subgroups with “extreme” and “moderate” frequent visits that were similar in Ontario, Alberta, and B.C. with distinct patterns of ED presentation. That a greater proportion of patients in the “extreme” subgroup were discharged or left against medical advice, and a smaller proportion were admitted following individual ED visits may reflect underlying factors related to patient illness, socioeconomic barriers, and challenging healthcare experiences. Such factors may include untreated withdrawal, socioeconomic barriers to staying in hospital, and healthcare provider difficulties with communication or feelings of futility when treating these patients. Patients in “extreme” subgroups demonstrated a higher mortality than those in “moderate” subgroups (9.3% and 6.6%), which was higher than mortality we observed in our comparison cohort of people with non-frequent ED utilization and substance use (4.3%). Interpreted in the context of low Charlson Comorbidity Indices, substance use and frequent ED visits appear to confer an increased risk of mortality unrelated to other medical conditions (e.g., this could reflect complications related to substance use itself, and/or lifestyle associated with substance use). Our identified “extreme” and “moderate” subgroups’ mortality was lower than that of our comparison cohort with frequent ED visits and no substance use (10.4%), an intuitive finding given that the latter comprised older patients who likely had additional clinical complexity.

### Comparison to previous studies

Our findings align with previous studies indicating high healthcare utilization and mortality among people with frequent ED visits and substance use [13, 14].

Our analysis corroborates previous work demonstrating that data-driven cluster analysis can discriminate subgroups of people who visit EDs frequently, which are comparable across Canadian provinces [14, 46]. The 6.6% and 9.3% 365-day mortality we identified in “moderate” and “extreme” subgroups in B.C.were comparable to 4.7% to 8.8% mortality reported among people who made alcohol-related ED visits with increasing frequency in Ontario [13]. Our finding that many patients leave against medical advice, particularly in the “extreme” subgroup, also aligns with previous work.

### Strengths and limitations

Our access to comprehensive population-level data in three jurisdictions allowed us the unique opportunity to characterize and compare people with frequent ED visits and substance use inter-provincially.

Our study is limited by differential data availability among provinces. We had access to physician billing (MSP) in B.C. only, and included this information along with DAD and NACRS data to comprehensively identify healthcare contact for substance use. However, since MSP includes both in-hospital and community services, its inclusion may have made our B.C. cohort different from patients in Ontario and Alberta. Similarly, mortality data were only available in B.C., and HMHDB only in Ontario and Alberta limiting comparability. Second, our study did not have access to data on important health determinants (e.g., employment, housing, education, Indigenous status, ethnicity). Furthermore, homelessness was only documented for patients with a HMHDB-reported admission, and is unvalidated with unclear accuracy and reliability. Nevertheless, our linked province-level databases allowed a broad-reaching characterization with the information available. Third, due to delays to data access and linkage, 2015/16 is our most recent data year. The evolving opioid overdose epidemic would likely influence results more recently. Nonetheless, characterization until 2015/16 offers an important understanding of our cohort at the beginning of the opioid overdose epidemic, and reveals patterns remaining relevant today. Finally, our ICD10-CA-based substance use definition for cohort identification is unvalidated. We adopted a CIHI definition to align our analysis with a national standard, however CIHI’s definition includes codes that may not select for our intended population (e.g., accidental poisonings).

### Clinical implications

Although our analyses are exploratory and did not identify causal factors underlying frequent ED visits, we hypothesize that recurrent ED visits by our patient cohort may suggest gaps in access to evidence-based substance use disorder management and mental health care in EDs and communities [47], particularly for the “extreme” subgroup. For instance, patients may present to EDs when their substance use-related needs are not met elsewhere, such as when primary care physicians are uncomfortable with substance use management and/or unable to liaise with addiction specialists. Or, ED physicians may not know which treatments are available and/or be unable to offer timely follow-up, leading to repeat ED presentations. Alternatively, in light of high overall levels of healthcare system encounters (e.g., ED visits, hospitalizations, visits to family physicians) our analyses may indicate that patients in our cohort may have undergone appropriate screening and/or have been offered first-line and additional treatments for substance use disorder at some point, but for a variety of reasons may not have benefited from these programs. Our findings suggest a need to explore whether improving access to or uptake of first-line and additional treatments for substance use disorder (e.g., managed alcohol programs), and strengthening continuity with primary and mental health care could benefit our patient cohort. A prevalence of gastrointestinal and liver-related pathology among our cohort, particularly in the “moderate” subgroup, suggests that screening for drug and alcohol use-related complications may be beneficial. Our analysis identified similar subgroups in Ontario, Alberta, and B.C., demonstrating generalizability across provinces. These province-specific characterizations suggest that clinical characteristics of our patient cohort and potential interventions may be applicable to multiple Canadian and non-Canadian jurisdictions. Our analyses suggest that healthcare providers, policy makers, and health planners in multiple jurisdictions may collaboratively plan common approaches to address the needs of comparable patient populations.

Our findings must be interpreted in light of evidence supporting the effectiveness of interventions targeted towards people with frequent ED visits. For instance, interventions like case management have been shown to reduce numbers of ED visits, and may produce overall cost savings [48, 49]. Substance and alcohol use presentations are among the factors associated with chronic high ED utilization among people with frequent ED visits, highlighting the clinical importance of the overlap between these two populations [50, 51]. Increasing recognition of multiple factors associated with long-term frequent ED utilization (e.g., substance use, homelessness, mental health issues) suggest that screening interventions could enable the early detection of patients at risk and the initiation of preventative interventions that address underlying unmet needs driving high utilization [50, 51]. ED visits may be the optimal points of healthcare contact to engage patients with harmful substance use in preventive intervention, and these opportunities are not always recognized [52]. Evidence and guidelines support that ED-based substance use screening and interventions such as brief motivational interviewing for patients with harmful alcohol use, and buprenorphine/naloxone and take-home naloxone for patients with opioid use disorder, can decrease harmful substance use and improve engagement in addictions care [16, 17, 53, 54]. Taken in context of the existing literature, our analyses emphasize the importance of recognizing the confluence of substance use and frequent ED utilization as conferring high risk particularly when ED visits are very high, and therefore that these factors should be considered a potential basis of targeted screening and interventions to mitigate future harm.

### Research implications

More research is needed to better understand differences between “extreme” and “moderate” subgroups of patients with frequent ED visits and substance use, and drivers of healthcare utilization and outcomes. This research should specifically engage patient subgroups to better understand unmet needs.

## Conclusions

Our study identified unique subgroups of people with substance use who make “extreme” and “moderate” frequent ED visits. Subgroups had similar utilization patterns and clinical characteristics in Ontario, Alberta, and B.C., suggesting that characteristics and potential interventions may be generalizable across multiple jurisdictions. The confluence of frequent ED utilization and substance use appeared to confer increased mortality risk, particularly among subgroups with extremely frequent ED visits. In the context of existing evidence supporting the effectiveness of ED-initiated interventions for patients with substance use, our data indicate a potential role for targeted screening and intervention based on frequency of ED use. Our findings suggest a need to explore whether improving access to and uptake of evidence-based substance use disorder management in EDs, and strengthening continuity with primary and mental health care, may benefit our target patient cohort.

## Supplementary Information

**Additional file 1: Supplementary Table 1.** Summary of diagnostic codes for substance use-related presentations. List of diagnostic codes used to define substance use categories in study cohort definition**. a. Supplementary Table 1.1.** Summary of ICD-10-CA codes for substance use categories. List of ICD-10-CA diagnostic codes used to define substance use categories. **b. Supplementary Table 1.2.** Summary of ICD-9 codes for substance use categories. List of ICD-9 diagnostic codes used to define substance use categories **Supplementary Table 2.** Variable checklist for each database. Summary of databases used to characterize study cohort (NACRS, DAD, HMHDB, MSP, Pharmanet, Vital Events and Statistics), and summary of variables characterized within each database**. Supplementary Table 3.** Pseudo F values for number of subgroups for Ontario, from 1-10. Summary of subgroup numbers and associated pseudo F values used for cluster analysis in Ontario. **Supplementary Table 4.** Pseudo F values for number of subgroups for Alberta, from 1-10. Summary of subgroup numbers and associated pseudo F values used for cluster analysis in Alberta**. Supplementary Table 5.** Pseudo F values for number of subgroups for B.C., from 1-10. Summary of subgroup numbers and associated pseudo F values used for cluster analysis in B.C.** Supplementary Figure 1.** Visual Representation of Subgroups for Ontario. Graphical representation of clustering variables used to define subgroups in Ontario**. Supplementary Figure 2.** Visual Representation of Subgroups for Alberta. Graphical representation of clustering variables used to define subgroups in Alberta**. Supplementary Figure 3.** Visual Representation of Subgroups for B.C. Graphical representation of clustering variables used to define subgroups in B.C.** Supplementary Table 6.** Demographic and healthcare utilization characteristics of subgroups of people with frequent ED visits (top 10%) and substance use in Ontario, Alberta, and B.C. (April 1^st^, 2014 to March 31^st^, 2015). Detailed summary of subgroups’ demographic and healthcare utilization characteristics to complement main study Tables.

## Data Availability

The datasets generated and analyzed during the current study are not publicly available due to data stewardship and access standards maintained by Population Data BC and the Canadian Institute for Health Information to ensure that data security and privacy are upheld. These datasets are available from Population Data BC and the Canadian Institute for Health Information via standard data access request processes (https://www.popdata.bc.ca/data_access/requirements; https://www.cihi.ca/en/access-data-and-reports/make-a-data-request

## Abbreviations

B.C.: British Columbia
CIHI: Canadian Institute of Health Information
CTAS: Canadian Triage and Acuity Scale
DAD: Discharge Abstract Database
ED: Emergency department
HMDB: Hospital Morbidity Database
HMHDB: Hospital Mental Health Database
HMHS: Hospital Mental Health Survey
ICD-10-CA: International Statistical Classification of Diseases and Related Health Problems 10th Revision
MSP: Medical Services Plan
NACRS: National Ambulatory Care Reporting System
OMHRS: Ontario Mental Health Reporting System
PopData: Population Data BC
UBC: University of British Columbia
