# Supplementary material for: Characterizing people with frequent emergency department visits and substance use: a retrospective cohort study of linked administrative data in Ontario, Alberta, and B.C., Canada
Source: BMC Emerg Med. 2022 Jul 14;22:127. doi: 10.1186/s12873-022-00673-x (PMC9281237; doi:10.1186/s12873-022-00673-x)
Supplement: Supplementary file 1 — Additional file 1: Supplementary Table 1. Summary of diagnostic codes for substance use-related presentations. List of diagnostic codes used to define substance use categories in study cohort definition. a. Supplementary Table 1.1. Summary of ICD-10-CA codes for substance use categories. List of ICD-10-CA diagnostic codes used to define substance use categories. b. Supplementary Table 1.2. Summary of ICD-9 codes for substance use categories. List of ICD-9 diagnostic codes used to define substance use categories Supplementary Table 2. Variable checklist for each database. Summary of databases used to characterize study cohort (NACRS, DAD, HMHDB, MSP, Pharmanet, Vital Events and Statistics), and summary of variables characterized within each database. Supplementary Table 3. Pseudo F values for number of subgroups for Ontario, from 1-10. Summary of subgroup numbers and associated pseudo F values used for cluster analysis in Ontario. Supplementary Table 4. Pseudo F values for number of subgroups for Alberta, from 1-10. Summary of subgroup numbers and associated pseudo F values used for cluster analysis in Alberta. Supplementary Table 5. Pseudo F values for number of subgroups for B.C., from 1-10. Summary of subgroup numbers and associated pseudo F values used for cluster analysis in B.C. Supplementary Figure 1. Visual Representation of Subgroups for Ontario. Graphical representation of clustering variables used to define subgroups in Ontario. Supplementary Figure 2. Visual Representation of Subgroups for Alberta. Graphical representation of clustering variables used to define subgroups in Alberta. Supplementary Figure 3. Visual Representation of Subgroups for B.C. Graphical representation of clustering variables used to define subgroups in B.C. Supplementary Table 6. Demographic and healthcare utilization characteristics of subgroups of people with frequent ED visits (top 10%) and substance use in Ontario, Alberta, and B.C. (April 1st, 2014 to March 31st, 2015). Detaile [file 12873_2022_673_MOESM1_ESM.docx]

**Supplementary Table 1. Summary of diagnostic codes for substance use-related presentations**

**Supplementary Table 1.1. Summary of ICD-10-CA codes for substance use categories**

| **Substance use category** | **ICD-10-CA codes** | **ICD-10-CA Description** |
| --- | --- | --- |
| Alcohol | E244 | Alcohol-induced pseudo-Cushing’s syndrome |
|  | F10.– | Mental and behavioural disorders due to use of alcohol |
|  | G312 | Degeneration of nervous system due to alcohol |
|  | G621 | Alcoholic polyneuropathy |
|  | G721 | Alcoholic myopathy |
|  | I426 | Alcoholic cardiomyopathy |
|  | K292 | Alcoholic gastritis |
|  | K70.– | Alcoholic liver disease |
|  | K852 | Alcohol-induced acute pancreatitis |
|  | K860 | Alcohol-induced chronic pancreatitis |
|  | O354– | Maternal care for (suspected) damage to fetus from alcohol |
|  | Q860 | Fetal alcohol syndrome (dysmorphic) |
|  | R780 | Finding of alcohol in blood |
|  | T51.– | Toxic effects of alcohol |
|  | X45 | Accidental poisoning by and exposure to alcohol |
|  | X65 | Intentional self-poisoning by and exposure to alcohol |
|  | Y15 | Poisoning by and exposure to alcohol, undetermined intent |
| Opioids | F11.– | Mental and behavioural disorders due to use of opioids |
|  | T400 | Poisoning by opium |
|  | T401 | Poisoning by heroin |
|  | T402 | Poisoning by other opioids |
|  | T403 | Poisoning by methadone |
|  | T404 | Poisoning by other synthetic narcotics |
|  | T406 | Poisoning by other and unspecified narcotics |
| Stimulants, cocaine, psychoactive and hallucinogens-related | F15.– | Mental and behavioural disorders due to use of other stimulants, including caffeine |
|  | T436 | Poisoning by other stimulants and amphetamines |
|  | F14.– | Mental and behavioural disorders due to use of cocaine |
|  | T405 | Poisoning by cocaine |
|  | F19.– | Mental and behavioural disorders due to use of multiple drug use and use of other psychoactive substances |
|  | T438 | Poisoning by other psychotropic drugs, not elsewhere classified |
|  | T439 | Poisoning by psychotropic drug, unspecified |
|  | X41 (when coded without T42–T43) | Accidental poisoning by and exposure to antiepileptic, sedative-hypnotic, antiparkinsonism and psychotropic drugs, not elsewhere classified |
|  | X61 (when coded without T42–T43) | Intentional self-poisoning by and exposure to antiepileptic, sedative-hypnotic, antiparkinsonism and psychotropic drugs, not elsewhere classified |
|  | Y11 (when coded without T42–T43) | Poisoning by and exposure to antiepileptic, sedative-hypnotic, antiparkinsonism and psychotropic drugs, not elsewhere classified, undetermined intent |
|  | Y12 (when coded without T40.–) | Poisoning by and exposure to narcotics and psychodysleptics, [hallucinogens], not elsewhere classified, undetermined intent |
|  | F16.– | Mental and behavioural disorders due to use of hallucinogens |
|  | T408 | Poisoning by lysergide [LSD] |
|  | T409 | Poisoning by other and unspecified psychodysleptics [hallucinogens] |
|  | X42 (when coded without T40.–) | Accidental poisoning by and exposure to narcotics and psychodysleptics [hallucinogens], not elsewhere classified |
|  | X62 (when coded without T40.–) | Intentional self-poisoning by and exposure to narcotics and psychodysleptics [hallucinogens], not elsewhere classified |
| Other substance use | F12.– | Mental and behavioural disorders due to use of cannabinoids |
|  | T407 | Poisoning by cannabis (derivatives) |
|  | F13.– | Mental and behavioural disorders due to use of sedatives or hypnotics |
|  | T423 | Poisoning by barbiturates |
|  | T424 | Poisoning by benzodiazepines |
|  | T426 | Poisoning by other antiepileptic and sedative-hypnotic drugs |
|  | T427 | Poisoning by antiepileptic and sedative-hypnotic drugs, unspecified |
|  | F18.– | Mental and behavioural disorders due to use of volatile solvents |
|  | F55 | Abuse of non-dependence-producing substances |

**Supplementary Table 1.2. Summary of ICD-9 codes for substance use categories**

| **Substance use category** | **ICD-9 codes** | **ICD-9 Description** |
| --- | --- | --- |
| Alcohol | 291.0 | Alcohol intoxication delirium or withdrawal delirium |
|  | 291.1 | Alcohol-induced major neurocognitive disorder, amnestic confabulatory type |
|  | 291.2 | Alcohol-induced major neurocognitive disorder, nonamnestic confabulatory type |
|  | 291.81 | Alcohol withdrawal |
|  | 291.82 | Alcohol-induced sleep disorder |
|  | 291.89 | Alcohol-induced anxiety disorder, bipolar and related disorder, depressive disorder, mild neurocognitive disorder or sexual dysfunction |
|  | 291.9 | Alcohol-induced psychotic disorder or unspecified alcohol-related disorder |
|  | 303.00  303.0– | Alcohol intoxication |
|  | 303.90  303.9– | Alcohol use disorder, moderate/severe |
|  | 305.00  305.0– | Alcohol use disorder, mild |
| Opioids | 304.00  304.0– | Opioid use disorder, moderate or severe |
|  | 305.50  305.5– | Opioid use disorder, mild |
| Stimulants, cocaine, psychoactive and hallucinogens-related | 304.20  304.2– | Cocaine use disorder, moderate or severe |
|  | 304.40  304.4– | Amphetamine-type substance use disorder, moderate or severe |
|  | 304.50  304.5– | Other hallucinogen use disorder, moderate or severe |
|  | 305.30  305.3– | Other hallucinogen use disorder, mild |
|  | 305.60  305.6– | Cocaine use disorder, mild |
|  | 305.70  305.7– | Amphetamine-type substance use disorder, mild |
|  | 304.60  304.6– | Inhalants and phencyclidine use disorder, moderate or severe |
| Other substance use | 304.10  304.1– | Sedative, hypnotic or anxiolytic use disorder, moderate or severe |
|  | 304.30  304.3– | Cannabis use disorder, moderate or severe |
|  | 305.20  305.2– | Cannabis use disorder, mild |
|  | 305.40  305.4– | Sedative, hypnotic or anxiolytic use disorder, mild |
|  | 292.0 | Substance withdrawal |
|  | 292.81 | Substance/medication-induced intoxication delirium |
|  | 292.82 | Substance-induced major neurocognitive disorder |
|  | 292.84 | Substance-induced depressive; substance-induced bipolar and related disorder |
|  | 292.85 | Substance-induced sleep disorder |
|  | 292.89 | Substance-induced: anxiety disorder, mild neurocognitive disorder, obsessive-compulsive and related disorder, sexual dysfunction, intoxication, other (or unknown) |
|  | 292.9 | Substance-induced psychotic disorder; unspecified substance-related disorder |
|  | 304.90  304.9– | Other (or unknown) substance use disorder, moderate or severe |
|  | 305.90  305.9– | Other (or unknown) substance use disorder, mild |

**Supplementary Table 2. Variable checklist for each database**

| Database | Variables | Definition |
| --- | --- | --- |
| National Ambulatory Care Reporting System (NACRS)-Patient Level | Number of patients | Count of patients |
|  | Gender | Original data |
|  | Age | Original data |
|  | Number of ED visits per patient | Count of ED visits individually |
|  | Rural/urban | Derived from the truncated postal code. Rural residence had “0” in the second position of the truncated postal code |
|  |  |  |
| National Ambulatory Care Reporting System (NACRS)-Visit Level | Number of ED visits | Count of ED visits |
|  | Arrive by ambulance | Original data |
|  | Triage level | Original data |
|  | Visit disposition | Original data |
|  | ICD-10-CA ED diagnosis chapters | Classified primary ED diagnosis into ICD-10-CA diagnosis chapter |
|  | Top 5 main problem | Top 5 primary ED diagnosis with ICD-10-CA code |
|  |  |  |
| Hospitalization Characteristics (DAD) | Number of patients | Count of patients |
|  | Gender | Original data |
|  | Age | Original data |
|  | Number of admissions per patient | Count of admissions individually |
|  | Number of admissions | Count of admissions |
|  | Total length of stay (days) | Original data |
|  | Disposition | Original data |
|  | ICD-10-CA primary diagnosis chapters | Classified the most responsible diagnosis for the patient during hospitalization into ICD-10-CA diagnosis chapter |
|  | Top 5 diagnosis | Top 5 the most responsible diagnosis for the patient during hospitalization with ICD-10-CA code |
|  |  |  |
| Mental Health Hospitalization Characteristics (HMHDB) (ON and AB) | Number of patients | Count of patients |
|  | Age | Original data |
|  | Number of mental health admissions per person | Count of mental health admissions individually |
|  | Gender | Original data |
|  | Homelessness | Original data: indicates whether a person was homeless on admission |
|  | Number of mental health admissions | Count of mental health admissions |
|  | Data source | Original data: indicates the original data source for the record |
|  | Length of stay at hospital (days) | Original data: the total number of days the person was hospitalized. |
|  | Discharge disposition | Original data: broad mental health category based on the most responsible separation diagnosis code |
|  | Diagnosis category | Original data |
|  |  |  |
| Medical service billing (MSP) dataset (B.C. only) | Number of patients | Count of patients |
|  | Total number of any MSP claims | Count of any MSP claims |
|  | Claim specialty group | Original data |
|  | MSP billing-related diagnosis (ICD-9) chapters | Classified the MSP billing-related diganosis into ICD-9 diagnosis chapter |
|  | Service location | Original data |
|  | Number of individual MSP claims | Count of MSP claims individually |
|  | Number of patients with general practice (GP) claim | Count of patients with GP claims |
|  | Number of GP claim per patient | Count of MSP GP claims divided by the total number of patients |
|  | Majority source of care | Identify whether ≥50% of patients’ services were provided by a single general practitioner, among patients who received ≥3 family doctor services in one year |
|  | Total number of GP claims | Count of MSP GP claims |
|  |  |  |
| Medication (Pharmanet) dataset (B.C. only) | Number of patients | Count of patients |
|  | Total number of entries for all medications | Count of medications |
|  | Number of drugs per individual by generic name | Count of drugs based on generic name (original data) individually |
|  | Number of drugs per individual by generic name | Count of drugs based on DINPIN (original data) individually |
|  | Top 10 American Hospital Formulary Service 1^st^ tier classification | Original data: classified drugs based on American Hospital Formulary Service classification (AHFS) classification system, 1^st^ tier and select top 10 |
|  |  |  |
| Vital Events and Statistics Deaths dataset (B.C. only) | Number of deaths | Count of deaths |
|  | Age at death | Original data |
|  | Number of ED visits among deaths | Count of ED visits among deaths |
|  | Days between last ED visit and death | Count days between last ED visit date and death date |
|  | Last ED visit ICD-10-CA diagnosis chapters among deaths | Last ED visit diagnosis classified into ICD-10-CA diagnosis chapters |
|  | Top 5 death cause ICD-10 code | Original data: ICD-10-CA code for death cause and select top 5 |

Footnote: Drug identification numbers (DINs) are Health Canada-assigned classifications for all drugs marketed in Canada (<https://www.canada.ca/en/health-canada/services/drugs-health-products/drug-products/fact-sheets/drug-identification-number.html>).

Product identification numbers (PINs) are an additional classification in B.C.’s PharmaCare system for certain drug products (<https://www2.gov.bc.ca/gov/content/health/practitioner-professional-resources/pharmacare/pharmacies/product-identification-numbers>).

**Supplementary Table 3. Pseudo F values for number of subgroups for Ontario, from 1-10**

| K Means subgroup number | 1 | 2 | 3 | 4 | 5 | 6 | 7 | 8 | 9 | 10 |
| --- | --- | --- | --- | --- | --- | --- | --- | --- | --- | --- |
| W | 195890 | 169401.2 | 152148.7 | 135691.3 | 123384.2 | 113045.4 | 104209.3 | 95567.5 | 87910.0 | 83308.9 |
| Pseudo F | Inf | 3062.9 | 2815.5 | 2896.4 | 2877.2 | 2870.4 | 2871.4 | 2936.6 | 3006.4 | 2940.0 |

**Footnote:** Two subgroups were chosen based on optimization of the pseudo-F statistic value, which compares between-cluster to within-cluster sum-of-squares.

**Supplementary Table 4. Pseudo F values for number of subgroups for Alberta, from 1-10**

| K Means subgroup number | 1 | 2 | 3 | 4 | 5 | 6 | 7 | 8 | 9 | 10 |
| --- | --- | --- | --- | --- | --- | --- | --- | --- | --- | --- |
| W | 76870.0 | 66418.7 | 59992.5 | 54489.0 | 49100.0 | 43990.6 | 40799.4 | 37152.8 | 34367.0 | 32256.5 |
| Pseudo F | Inf | 1209.4 | 1081.0 | 1052.0 | 1086.3 | 1148.3 | 1131.8 | 1172.9 | 1187.1 | 1179.9 |

**Footnote:** Two subgroups were chosen based on optimization of the pseudo-F statistic value, which compares between-cluster to within-cluster sum-of-squares.

**Supplementary Table 5. Pseudo F values for number of subgroups for B.C., from 1-10**

| K Means subgroup number | 1 | 2 | 3 | 4 | 5 | 6 | 7 | 8 | 9 | 10 |
| --- | --- | --- | --- | --- | --- | --- | --- | --- | --- | --- |
| W | 94020 | 80939.1 | 73597.77 | 64630.93 | 58493.51 | 53170.3 | 46626.69 | 41243.71 | 37622.54 | 34368.47 |
| Pseudo F | -Inf | 1519.33 | 1304.176 | 1424.642 | 1426.987 | 1443.906 | 1591.747 | 1717.433 | 1760.241 | 1811.436 |

**Footnote:** Two subgroups were chosen based on optimization of the pseudo-F statistic value, which compares between-cluster to within-cluster sum-of-squares.

**Supplementary Figure 1. Visual Representation of Subgroups for Ontario**


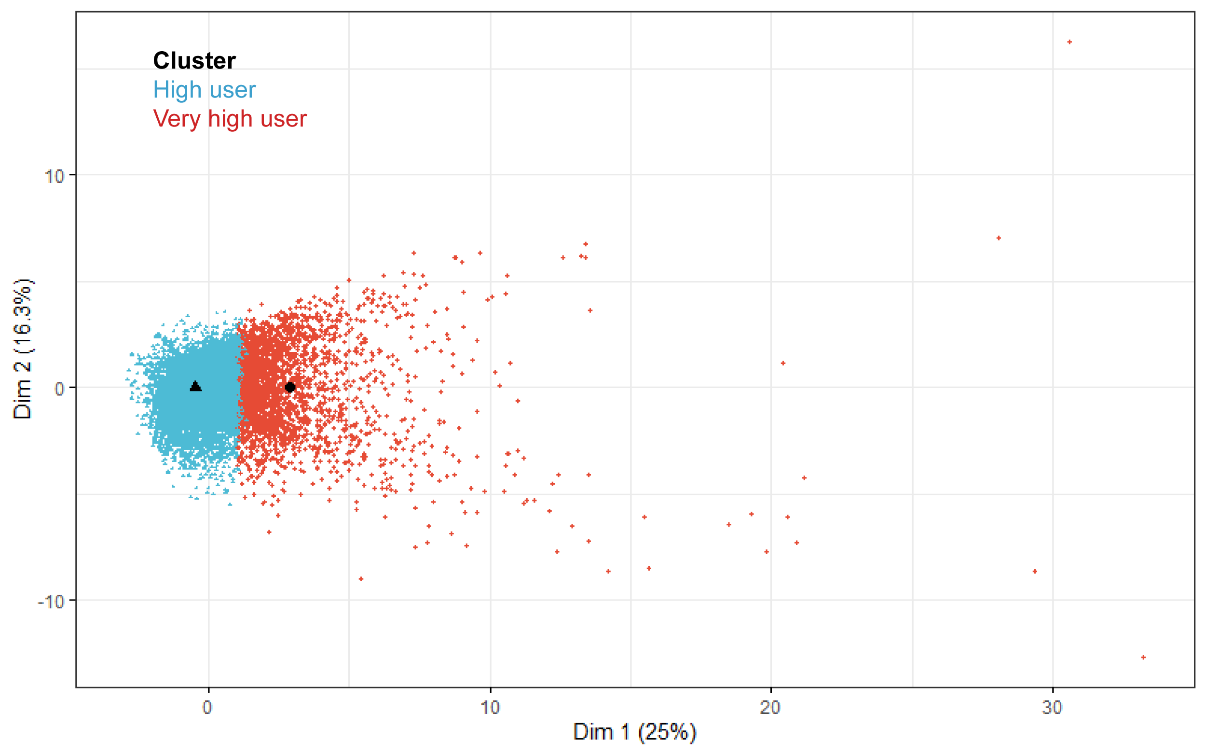


**
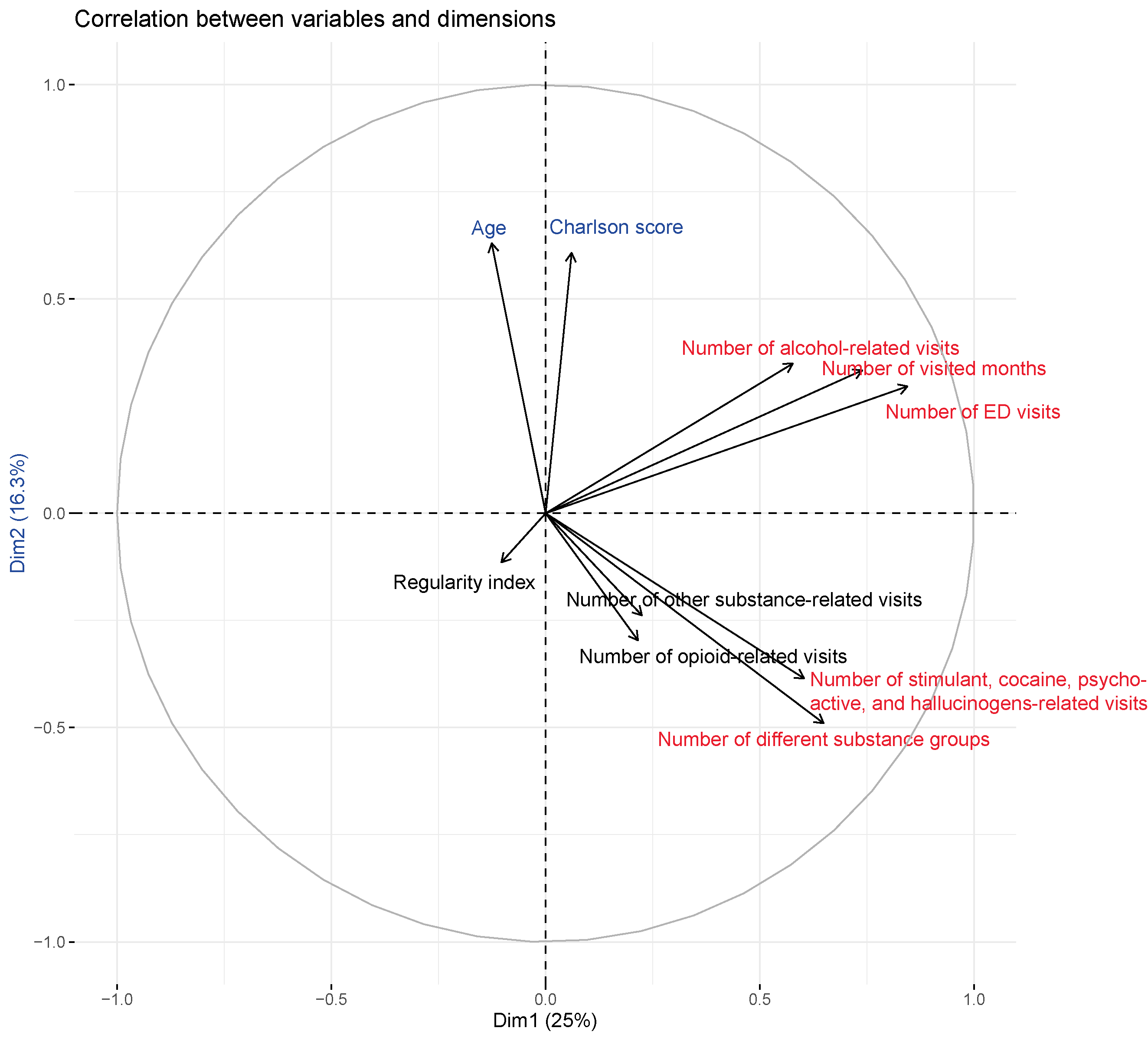
**

Footnote: Black markers indicate the cluster mean for each group.

We were interested in a graphical representation of our subgroups, but doing so required a reduction of the dimensionality of the data so that it could be represented on a graph. One approach to this is to apply a principal components-type analysis after clustering, and then plotting the most important variables against the second most important variables (these are Dim1 and Dim2). Supplementary Figure 1 shows clusters of samples in Ontario based on their similarity. Dimension 1 accounted for 25% of the information. Dimension 2 accounted for 16.3% of the information.

The plot above suggests that number of alcohol-related diagnoses, number of stimulants, cocaine, psychoactive and hallucinogens-related diagnoses, number of distinct substance groups, number of months that the patient visited an ED, and ED visit number had strong positive effects on Dimension 1. Regularity index had a strong negative effect on Dimension 1. Age at index, and Charlson comorbidity index had strong positive effects on Dimension 2.

**Supplementary Figure 2. Visual Representation of Subgroups for Alberta**


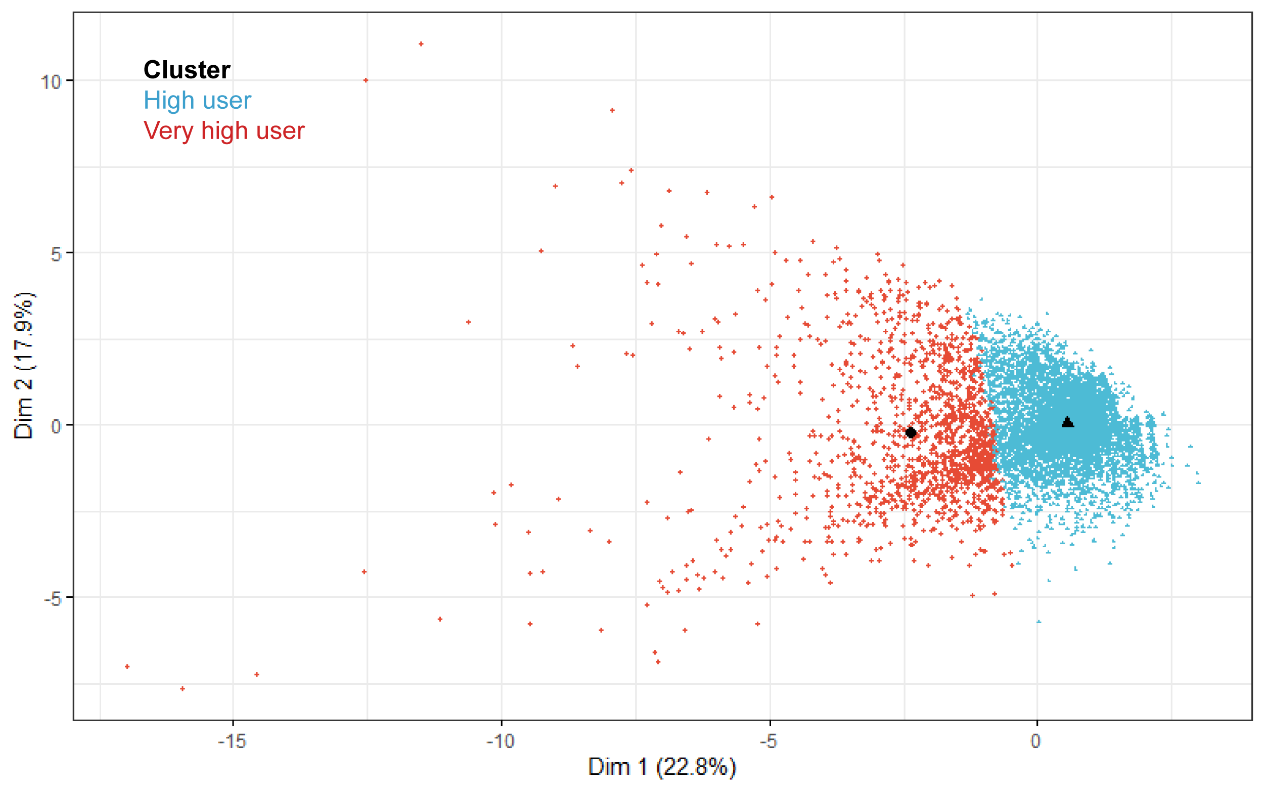


**
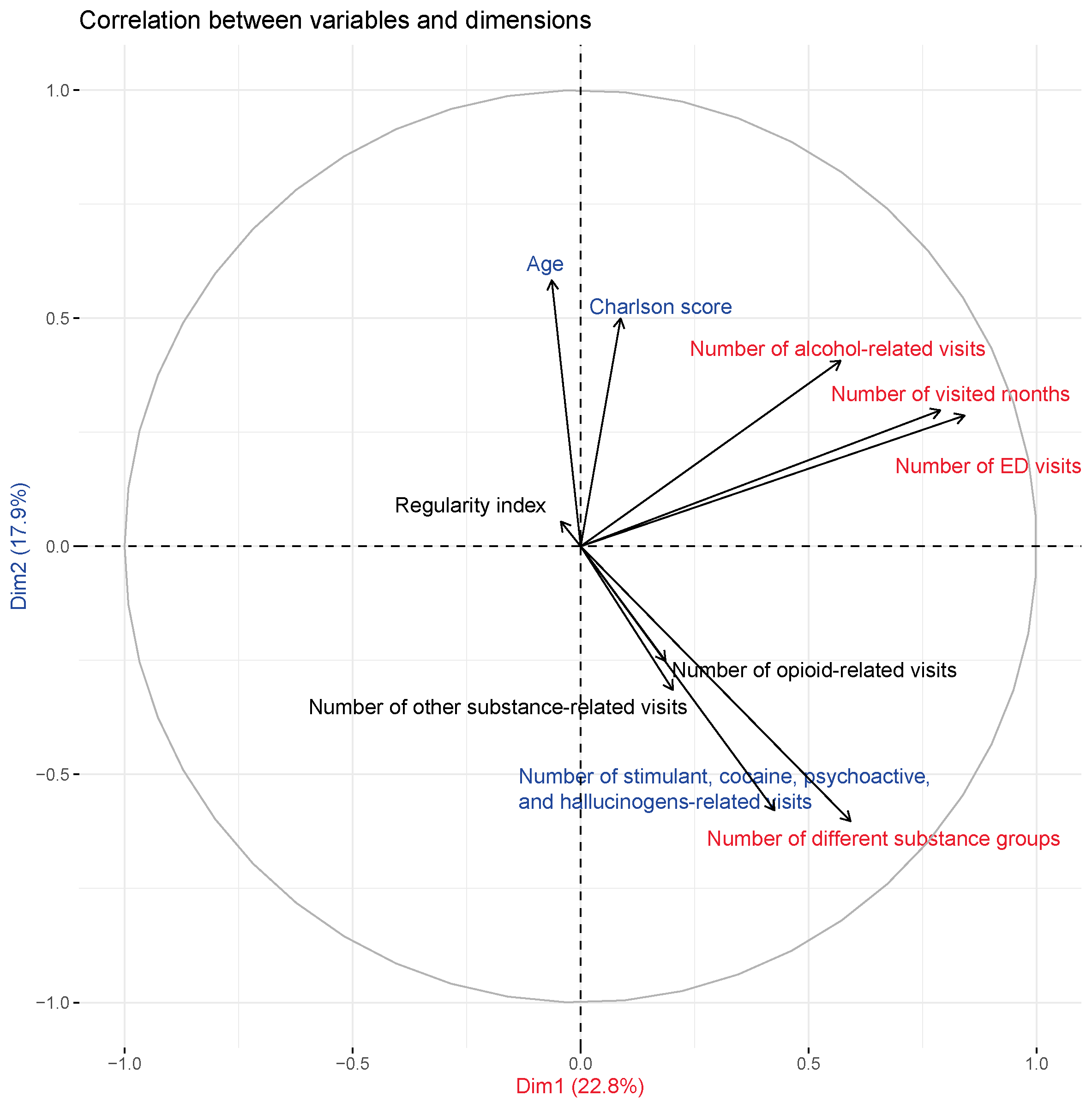
**

Footnote: Black markers indicate the cluster mean for each group.

We were interested in a graphical representation of our subgroups, but doing so required a reduction of the dimensionality of the data so that it could be represented on a graph. One approach to this is to apply a principal components-type analysis after clustering, and then plotting the most important variables against the second most important variables (these are Dim1 and Dim2). Supplementary Figure 2 shows clusters of samples in Alberta based on their similarity. Dimension 1 accounted for 22.8% of the information. Dimension 2 accounted for 17.9% of the information.

The plot above suggests that number of alcohol-related diagnoses, number of distinct substance groups, number of months that the patient visited an ED, and ED visit number had strong positive effects on Dimension 1. Regularity index had a strong negative effect on Dimension 1. Age at index, and Charlson comorbidity index had strong positive effects on Dimension 2. Number of stimulants, cocaine, psychoactive and hallucinogens-related diagnoses had strong negative effects on Dimension 2.

**Supplementary Figure 3. Visual Representation of Subgroups for B.C.**


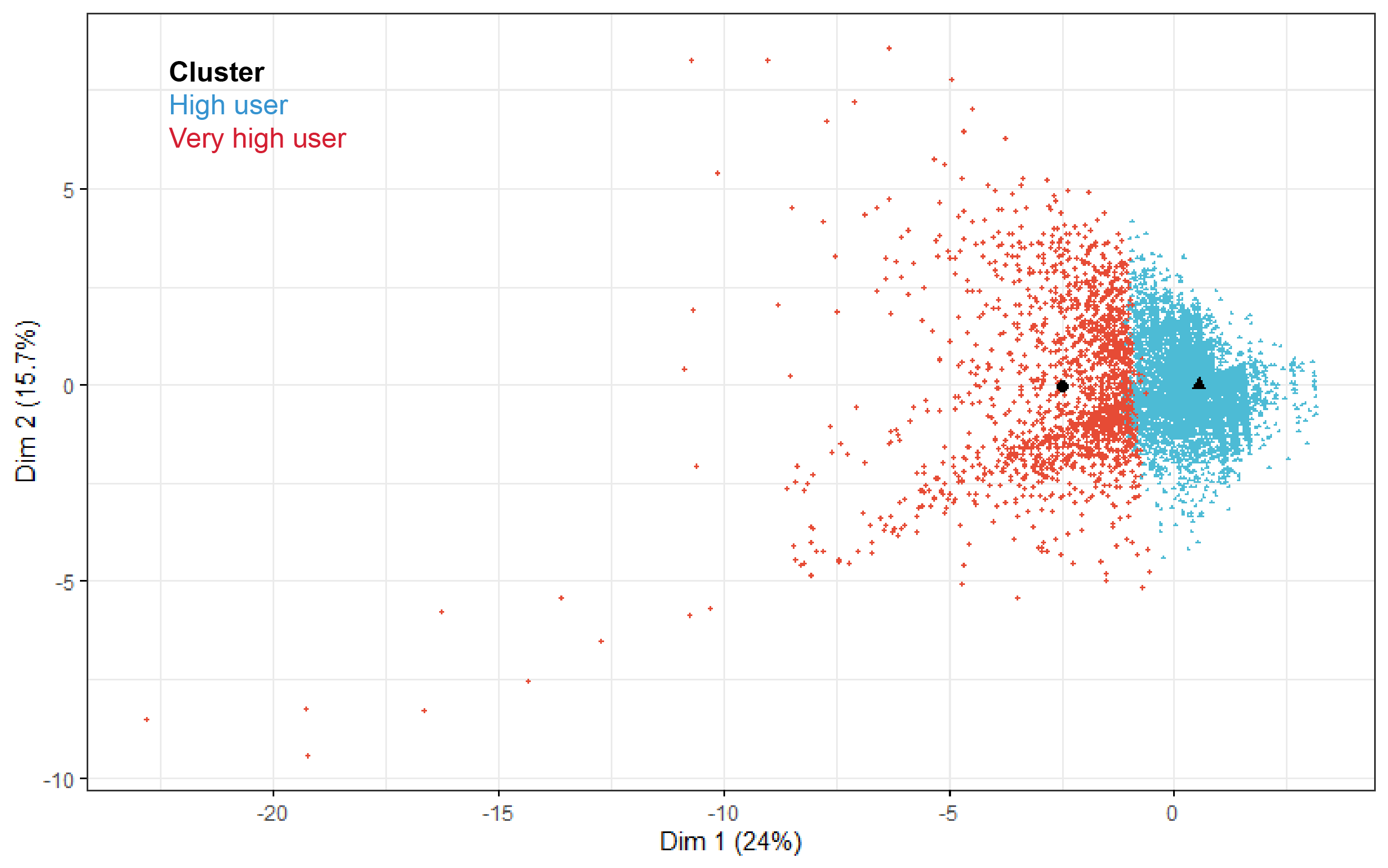


Footnote: Black markers indicate the cluster mean for each group.

Dim 1 and Dim 2 represent the first two dimensions in a principal component analysis. In order to visualize our cluster analysis, we used principal component analysis to project the initial data onto the first two principal components. The contribution of each variable to the first two dimensions is shown in the following table:

| Variables | Dim.1 | Dim.2 |
| --- | --- | --- |
| Age | 0.1% | 21.6% |
| Number of visits to the ED | 29.9% | 5.2% |
| Number of alcohol-related visits | 15.4% | 6.1% |
| Number of stimulants, cocaine, psychoactive and hallucinogens-related visits | 7.7% | 19.1% |
| Number of opioid-related visits | 2.7% | 13.1% |
| Number of other substance-related visits | 0.6% | 0.8% |
| Number of different substance groups | 14.0% | 18.6% |
| Weighted Charlson score | 1.1% | 11.5% |
| Number of months in the year visited ED | 27.3% | 4.0% |
| Regularity index | 1.2% | 0.0% |

**Supplementary Table 6. Demographic and healthcare utilization characteristics of subgroups of people with frequent ED visits (top 10%) and substance use in Ontario, Alberta, and B.C.** **(April 1^st^, 2014 to March 31^st^, 2015)**

| **Characteristics** | **Ontario** | | **Alberta** | | **British Columbia** | |
| --- | --- | --- | --- | --- | --- | --- |
|  | **Extreme (N=2,669)** | **Moderate (N=16,921)** | **Extreme (N=1,468)** | **Moderate (N=6,220)** | **Extreme (N=1,690)** | **Moderate (N=7,713)** |
| ***Subgroup Characteristics (All Clustering Variables)*** | | | | | | |
|  |  |  |  |  |  |  |
| **Age (years)** | | | | | | |
| Median (IQR) | 36 (27-49) | 40 (27-54) | 38 (29-49) | 37 (27-50) | 45 (33-53) | 44 (31-56) |
|  |  |  |  |  |  |  |
| **Total number of ED visits** | | | | | | |
| Median (IQR) | 19 (13-28) | 5 (4-8) | 19 (15-27) | 6 (5-9) | 13 (10-20) | 4 (3-6) |
|  |  |  |  |  |  |  |
| **Number of alcohol-related ED visits** | | | | | | |
| Median (IQR) | 1 (0-7) | 0 (0-1) | 2 (0-7) | 1 (0-2) | 1 (0-4) | 0 (0-1) |
|  |  |  |  |  |  |  |
| **Number of stimulants, cocaine, psychoactive and hallucinogens-related ED visits** | | | | | | |
| Median (IQR) | 1 (0-2) | 0 (0-0) | 0 (0-1) | 0 (0-0) | 0 (0-1) | 0 (0-0) |
|  |  |  |  |  |  |  |
| **Number of opioid-related visits** | | | | | | |
| Median (IQR) | 0 (0-1) | 0 (0-0) | 0 (0-0) | 0 (0-0) | 0 (0-0) | 0 (0-0) |
|  |  |  |  |  |  |  |
| **Number of other substance-related ED visits** | | | | | | |
| Median (IQR) | 0 (0-0) | 0 (0-0) | 0 (0-0) | 0 (0-0) | 0 (0-0) | 0 (0-0) |
|  |  |  |  |  |  |  |
| **Number of different substance groups for which patients made ED visits** | | | | | | |
| Median (IQR) | 2 (1-2) | 1 (1-1) | 1 (1-2) | 1 (1-1) | 1 (1-2) | 1 (0-1) |
|  |  |  |  |  |  |  |
| **Weighted Charlson Comorbidity Index** | | | | | | |
| Median (IQR) | 0 (0-0) | 0 (0-0) | 0 (0-0) | 0 (0-0) | 0 (0-0) | 0 (0-0) |
|  |  |  |  |  |  |  |
| **Number of months of the year that patents visited EDs** | | | | | | |
| Median (IQR) | 8 (7-10) | 4 (3-5) | 8 (7-10) | 4 (3-5) | 7 (6-9) | 3 (3-4) |
|  |  |  |  |  |  |  |
| **Regularity index of ED visits** | | | | | | |
| Median (IQR) | 0.2×10^-2^ (0.1×10^-2^ -0.5×10^-2^) | 0.5×10^-3^ (0.2×10^-3^ -1.4×10^-3^) | 0.2×10^-2^ (0.1×10^-2^ -0.4×10^-2^) | 0.5×10^-3^ (0.3×10^-3^ -1.1×10^-3^) | 1.5×10^-3^ (0.8×10^-3^ -3.1×10^-3^) | 0.5×10^-3^ (0.2×10^-3^ -1.5×10^-3^) |
|  |  |  |  |  |  |  |
| ***ED Visit Characteristics (NACRS)*** | | | | | | |
| **Number of ED visits (for any reason)** | **66,655** | **102,807** | **34,400** | **44,193** | **28,893** | **39,181** |
|  |  |  |  |  |  |  |
| **Triage level among ED visits, n (%)** |  |  |  |  |  |  |

| 1 (Resuscitation) | 1,019 (1.5) | 2,076 (2.0) | 452 (1.3) | 629 (1.4) | 276 (1.0) | 506 (1.3) |
| --- | --- | --- | --- | --- | --- | --- |
| 2 (Emergent) | 18,995 (28.5) | 30,633 (29.8) | 6,415 (18.6) | 8,647 (19.6) | 5,269 (18.2) | 8,239 (21) |
| 3 (Urgent) | 31,378 (47.1) | 47,046 (45.8) | 13,322 (38.7) | 17,561 (39.7) | 15,882 (55.0) | 20,148 (51.4) |
| 4 (Less-urgent) | 11,771 (17.7) | 18,477 (18.0) | 9,544 (27.7) | 11,894 (26.9) | 6,319 (21.9) | 8,751 (22.3) |
| 5 (Non-urgent) | 2,907 (4.4) | 3,839 (3.7) | 3,823 (11.1) | 4,116 (9.3) | 1,086 (3.8) | 1,444 (3.7) |
| 9 (Unknown) | 496 (0.7) | 629 (0.6) | 713 (2.1) | 1,221 (2.8) | 61 (0.2) | 93 (0.2) |
| NA | 89 (0.1) | 107 (0.1) | 131 (0.4) | 125 (0.3) | 0 (0.0) | 0 (0.0) |
|  |  |  |  |  |  |  |

| **ED diagnosis (ICD-10-CA) chapters among ED visits, n (%)** | | | | | | |
| --- | --- | --- | --- | --- | --- | --- |
| Chapter 1-Infectious and parasitic diseases (A00-B99) | 815 (1.2) | 1,713 (1.7) | 563 (1.6) | 804 (1.8) | 359 (1.2) | 556 (1.4) |
| Chapter 2-Neoplasms (C00-D48) | 44 (0.1) | 169 (0.2) | 24 (0.1) | 54 (0.1) | 21 (0.1) | 34 (0.1) |
| Chapter 3-Hematologic and immunologic diseases (D50-D89) | 85 (0.1) | 283 (0.3) | 43 (0.1) | 114 (0.3) | 33 (0.1) | 97 (0.2) |
| Chapter 4-Endocrine, nutritional and metabolic diseases (E00-E90) | 636 (1.0) | 1229 (1.2) | 408 (1.2) | 651 (1.5) | 271 (0.9) | 343 (0.9) |
| Chapter 5-Mental and behavioral disorders (F00-F99) | 27,788 (41.7) | 33,587 (32.7) | 10,834 (31.5) | 12,086 (27.3) | 7,144 (24.7) | 7,377 (18.8) |
| Chapter 6-Diseases of the nervous system (G00-G99) | 1,154 (1.7) | 1,517 (1.5) | 981 (2.9) | 771 (1.7) | 222 (0.8) | 302 (0.8) |
| Chapter 7&8-Diseases of the eye, adnexa, ear and mastoid process (H00-H95) | 307 (0.5) | 798 (0.8) | 227 (0.7) | 397 (0.9) | 127 (0.4) | 224 (0.6) |
| Chapter 9-Diseases of the circulatory system (I00-I99) | 530 (0.8) | 1,555 (1.5) | 221 (0.6) | 637 (1.4) | 303 (1.0) | 495 (1.3) |
| Chapter 10-Diseases of the respiratory system | 1,714 (2.6) | 3,710 (3.6) | 1,130 (3.3) | 1,745 (3.9) | 960 (3.3) | 1,106 (2.8) |
| Chapter 11-Diseases of the digestive system (K00-K93) | 2,457 (3.7) | 7,174 (7.0) | 1,861 (5.4) | 3,206 (7.3) | 1016 (3.5) | 1,505 (3.8) |
| Top 5 Chapter 11 Diagnosis |  |  |  |  |  |  |
|  | Constipation (K590):  282 (0.4) | Acute pancreatitis, unspecified (K859):  770 (0.7) | Periapical abscess without sinus (K047):  173 (0.5) | Alcoholic gastritis (K292):  364 (0.8) |  |  |
|  | Toothache NOS (K0887):  207 (0.3) | Gastrointestinal haemorrhage, unspecified (K922):  547 (0.5) | Alcoholic gastritis (K292):  147 (0.4) | Acute pancreatitis, unspecified (K859):  271 (0.6) |  |  |
|  | Gastrointestinal haemorrhage, unspecified (K922):  140 (0.2) | Hepatic failure, unspecified (K729):  473 (0.5) | Toothache NOS (K0887):  131 (0.4) | Gastritis, unspecified (K297):  199 (0.5) |  |  |
|  | Gastritis, unspecified (K297):  133 (0.2) | Constipation (K590):  381 (0.4) | Gastritis, unspecified (K297):  119 (0.3) | Gastrointestinal haemorrhage, unspecified (K922):  193 (0.4) |  |  |
|  | Acute pancreatitis, unspecified (K859):  133 (0.2) | Alcoholic cirrhosis of liver (K703):  378 (0.4) | Gastrointestinal haemorrhage, unspecified (K922):  103 (0.3) | Periapical abscess without sinus (K047):  160 (0.4) |  |  |
|  |  |  |  |  |  |  |
| Chapter 12-Dermatologic diseases (L00-L99) | 1,476 (2.2) | 2,775 (2.7) | 927 (2.7) | 1,309 (3.0) | 789 (2.7) | 1,290 (3.3) |
| Chapter 13- Diseases of the musculoskeletal system and connective tissue (M00-M99) | 3,018 (4.5) | 4,134 (4.0) | 1,777 (5.2) | 1,863 (4.2) | 1023 (3.5) | 1,312 (3.3) |
| Chapter 14-Diseases of the genitourinary system (N00-N99) | 967 (1.5) | 2,275 (2.2) | 570 (1.7) | 957 (2.2) | 485 (1.7) | 665 (1.7) |
| Chapter 15-Pregnancy, childbirth and the puerperium (O00-O99) | 148 (0.2) | 458 (0.4) | 153 (0.4) | 285 (0.6) | 14 (0.0) | 52 (0.1) |
| Chapter 16- Certain conditions originating in the perinatal period | 1 (0.0) | 1 (0.0) | 1 (0.0) | 0 (0.0) | 0 (0.0) | <5 (0.0) |
| Chapter 17 - Congenital malformations, deformations and chromosomal abnormalities | 8 (0.0) | 21 (0.0) | 15 (0.0) | 11 (0.0) | 0 (0.0) | 0 (0.0) |
| Chapter 18-Symptoms, signs and abnormal findings (R00-R99) | 11,862 (17.8) | 18,079 (17.6) | 5,385 (15.7) | 6,342 (14.4) | 4,591 (15.9) | 5,439 (13.9) |
| Chapter 19-Injury, poisoning and certain other consequences of external causes (S00-T98) | 7,608 (11.4) | 15,890 (15.5) | 5,426 (15.8) | 8,838 (20.0) | 4,700 (16.3) | 6,518 (16.6) |
| Chapter 21-Factors influencing health status and contact with health services (Z00-Z99) | 6,037 (9.1) | 7,439 (7.2) | 3,854 (11.2) | 4,123 (9.3) | 1,831 (6.3) | 1,903 (4.9) |
| NA | 0 (0.0) | 0 (0.0) | 0 (0.0) | 0 (0.0) | 5,004 (17.3) | 9,962 (25.4) |
|  |  |  |  |  |  |  |
| ***Hospitalization Characteristics (DAD)*** | | | | | | |
|  | **Extreme (N=2,669)** | **Moderate (N=16,921)** | **Extreme (N=1,468)** | **Moderate (N=6,220)** | **Extreme (N=1,690)** | **Moderate (N=7,713)** |
|  |  |  |  |  |  |  |
| **Number of admissions (for any reason)** | **4,498** | **13,648** | **3,647** | **6,981** | **5,499** | **10,446** |
|  |  |  |  |  |  |  |
| **Primary diagnosis ICD-10-CA chapters among admissions, n (%)** | | | | | | |
| Chapter 1-Certain infectious and parasitic diseases (A00-B99) | 154 (3.4) | 405 (3.0) | 110 (3.0) | 171 (2.4) | 143 (2.6) | 278 (2.7) |
| Chapter 2-Neoplasms (C00-D48) | 18 (0.4) | 104 (0.8) | 9 (0.2) | 32 (0.5) | 9 (0.2) | 68 (0.7) |
| Chapter 3-Diseases of the blood and blood-forming organs and certain disorders involving the immune mechanism (D50-D89) | 20 (0.4) | 100 (0.7) | 24 (0.7) | 43 (0.6) | 23 (0.4) | 54 (0.5) |
| Chapter 4-Endocrine, nutritional and metabolic diseases (E00-E90) | 164 (3.6) | 490 (3.6) | 90 (2.5) | 198 (2.8) | 179 (3.3) | 285 (2.7) |
| Chapter 5-Mental and behavioral disorders (F00-F99) | 1,106 (24.6) | 2,694 (19.7) | 1,596 (43.8) | 2,861 (41) | 2,812 (51.1) | 4,919 (47.1) |
| Chapter 6-Diseases of the nervous system (G00-G99) | 116 (2.6) | 344 (2.5) | 91 (2.5) | 113 (1.6) | 96 (1.7) | 141 (1.3) |
| Chapter 7-Diseases of the eye and adnexa (H00-H59) | 4 (0.1) | 10 (0.1) | 3 (0.1) | 11 (0.2) | 6 (0.1) | 6 (0.1) |
| Chapter 8-Diseases of the ear and mastoid process (H60-H95) | 3 (0.1) | 5 (0.0) | 4 (0.1) | 2 (0.0) | 0 (0.0) | 6 (0.1) |
| Chapter 9-Diseases of the circulatory system (I00-I99) | 181 (4) | 734 (5.4) | 67 (1.8) | 239 (3.4) | 169 (3.1) | 441 (4.2) |
| Chapter 10-Diseases of the respiratory system | 277 (6.2) | 813 (6.0) | 257 (7.0) | 360 (5.2) | 368 (6.7) | 535 (5.1) |
| Chapter 11-Diseases of the digestive system (K00-K93) | 629 (14.0) | 3,444 (25.2) | 479 (13.1) | 1224 (17.5) | 566 (10.3) | 1,240 (11.9) |
| Chapter 12-Diseases of the skin and subcutaneous tissue (L00-L99) | 145 (3.2) | 209 (1.5) | 101 (2.8) | 110 (1.6) | 96 (1.7) | 173 (1.7) |
| Chapter 13- Diseases of the musculoskeletal system and connective tissue (M00-M99) | 105 (2.3) | 232 (1.7) | 56 (1.5) | 95 (1.4) | 91 (1.7) | 206 (2.0) |
| Chapter 14-Diseases of the genitourinary system (N00-N99) | 88 (2.0) | 339 (2.5) | 67 (1.8) | 129 (1.8) | 90 (1.6) | 204 (2.0) |
| Chapter 15-Pregnancy, childbirth and the puerperium (O00-O99) | 30 (0.7) | 71 (0.5) | 34 (0.9) | 65 (0.9) | 13 (0.2) | 82 (0.8) |
| Chapter 17-Congenital malformations, deformations and chromosomal abnormalities (Q00-Q99) | 1 (0.0) | 4 (0.0) | 0 (0.0) | 3 (0.0) | 0 (0.0) | 7 (0.1) |
| Chapter 18-Symptoms, signs and abnormal clinical and laboratory findings, not elsewhere classified (R00-R99) | 467 (10.4) | 1,074 (7.9) | 207 (5.7) | 321 (4.6) | 373 (6.8) | 647 (6.2) |
| Chapter 19-Injury, poisoning and certain other consequences of external causes (S00-T98) | 934 (20.8) | 2,371 (17.4) | 412 (11.3) | 909 (13.0) | 426 (7.7) | 1,049 (10.0) |
| Chapter 21-Factors influencing health status and contact with health services (Z00-Z99) | 56 (1.2) | 205 (1.5) | 40 (1.1) | 95 (1.4) | 39 (0.7) | 105 (1.0) |
| NA | 0 (0.0) | 0 (0.0) | 0 (0.0) | 0 (0.0) | 0 (0.0) | 0 (0.0) |
|  |  |  |  |  |  |  |

| ***Mental Health Admission Characteristics (HMHDB)*** | | | | | | |
| --- | --- | --- | --- | --- | --- | --- |
|  | **Extreme (N=2,669)** | **Moderate (N=16,921)** | **Extreme (N=1,468)** | **Moderate (N=6,220)** | **Extreme (N=1,690)** | **Moderate (N=7,713)** |
| **Age of patients with at least one mental health admission** | | | | | | |
| Median (IQR) | 35 (27-48) | 41 (28-54) | 38 (29-49) | 39 (29-52) | NA | NA |
|  |  |  |  |  |  |  |
| **Sex among patients with at least one admission, n (%)** | | | | | | |
| Male | 858 (61.1) | 3,731 (61.6) | 432 (62.2) | 1142 (61.8) | NA | NA |
| Female | 546 (38.9) | 2,321 (38.3) | 263 (37.8) | 707 (38.2) | NA | NA |
| Others | 1 (0.1) | 2 (0.0) | 0 (0.0) | 0 (0.0) | NA | NA |
|  |  |  |  |  |  |  |
| **Number of mental health admissions (for any reason)** | **4,294** | **10,229** | **1,605** | **2,876** | NA | NA |
|  |  |  |  |  |  |  |
| **Length of stay (days) among mental health admissions** | | | | | | |
| Median (IQR) | 3 (1-8) | 4 (2-11) | 3 (1-6) | 4 (2-10) | NA | NA |
|  |  |  |  |  |  |  |
|  |  |  |  |  |  |  |
